# Norepinephrine links astrocytic activity to regulation of cortical state

**DOI:** 10.1038/s41593-023-01284-w

**Published:** 2023-03-30

**Authors:** Michael E. Reitman, Vincent Tse, Xuelong Mi, Drew D. Willoughby, Alba Peinado, Alexander Aivazidis, Bat-Erdene Myagmar, Paul C. Simpson, Omer A. Bayraktar, Guoqiang Yu, Kira E. Poskanzer

**Affiliations:** 1 Neuroscience Graduate Program, University of California, San Francisco, San Francisco, CA USA; 2 Department of Biochemistry & Biophysics, University of California, San Francisco, San Francisco, CA USA; 3 Bradley Department of Electrical and Computer Engineering, Virginia Polytechnic Institute and State University, Arlington, VA USA; 4 Wellcome Sanger Institute, Cambridge, UK; 5 Department of Medicine and Research Service, San Francisco Veterans Affairs Medical Center and Cardiovascular Research Institute, University of California, San Francisco, San Francisco, CA USA; 6 Kavli Institute for Fundamental Neuroscience, San Francisco, CA USA

**Keywords:** Astrocyte, Neural circuits

## Abstract

Cortical state, defined by population-level neuronal activity patterns, determines sensory perception. While arousal-associated neuromodulators—including norepinephrine (NE)—reduce cortical synchrony, how the cortex resynchronizes remains unknown. Furthermore, general mechanisms regulating cortical synchrony in the wake state are poorly understood. Using in vivo imaging and electrophysiology in mouse visual cortex, we describe a critical role for cortical astrocytes in circuit resynchronization. We characterize astrocytes’ calcium responses to changes in behavioral arousal and NE, and show that astrocytes signal when arousal-driven neuronal activity is reduced and bi-hemispheric cortical synchrony is increased. Using in vivo pharmacology, we uncover a paradoxical, synchronizing response to Adra1a receptor stimulation. We reconcile these results by demonstrating that astrocyte-specific deletion of *Adra1a* enhances arousal-driven neuronal activity, while impairing arousal-related cortical synchrony. Our findings demonstrate that astrocytic NE signaling acts as a distinct neuromodulatory pathway, regulating cortical state and linking arousal-associated desynchrony to cortical circuit resynchronization.

## Abstract (web summary)

The authors show that norepinephrine signaling to astrocytes in the mouse visual cortex acts as a separate neuromodulatory pathway that regulates the effects of arousal on neuronal activity and cortical state.

## Main

Patterns of neural activity in the awake cortex are variable^1^, ranging from states of highly synchronized neuronal activity during periods of low arousal, to states of desynchronized activity during high-arousal periods such as whisking^2^ and running^3^. These cortical states can be identified by electrophysiological activity: synchronized cortical states display greater low-frequency (LF) oscillations and reduced high-frequency (HF) oscillations than desynchronized states^4^. While HF oscillations are important for processing of incoming information^5^, LF oscillations during the wake state are less well characterized. Generally described within a 2–10 Hz range^2,6,7^, wakeful LF power differs substantively from cortical activity patterns during sleep^2^, indicating a unique role for synchronized cortical states in awake animals. One role of waking cortical synchrony may be to modulate sensory responses to external stimuli. Increased LF power is associated with reduced neuronal gain^6^ and broader tuning^8^ in sensory cortex, as well as diminished behavioral performance on sensory perception tasks^3,7,8^.

The mechanisms by which cortical state is regulated, and how the cortex becomes sensitized to external stimuli, are well studied. One central mechanism is signaling by norepinephrine (NE), which regulates a primary hallmark of behavioral arousal—pupil diameter^9^—and alters the firing properties of cortical neurons leading to desynchronized cortical activity^10,11^. However, while the desynchronizing effects of NE are well known, mechanisms that increase waking synchrony are less clear. A full understanding of mechanisms that regulate awake cortical synchrony will be necessary to explain fluctuations in perception and behavior.

Neurons are not the only NE-responsive cell types in the cortex^12,13^. Astrocytes, a non-neuronal cell type abundant throughout the cortex, respond to NE with robust calcium (Ca^2+^) signaling^14–19^. However, astrocyte Ca^2+^ has also been linked to increased cortical synchrony when NE signaling is low, including during sleep^20^ and under anesthesia^21^. In this Article, we aimed to resolve the disparity between an astrocytic role in sleep generation and their activation by NE and desynchronizing stimuli such as movement. We hypothesized that NE-specific astrocytic signaling might act as a mechanism that regulates NE-driven cortical desynchrony and restores cortical synchrony following changes in arousal.

Here we use in vivo, two-photon (2P) Ca^2+^ imaging to show that astrocytes in mouse visual cortex respond proportionally and with temporal specificity to changes in arousal and NE, positioning astrocytes as a local feedback mechanism to the effects of arousal. In agreement with this hypothesis, NE-driven astrocyte Ca^2+^ signaling occurs alongside reductions in arousal-associated neuronal activity. Using in vivo local field potential (LFP) recordings, we further show that arousal-driven astrocyte Ca^2+^ activity occurs at transitions from cortical desynchrony to synchrony, and that this relationship is dependent on NE signaling. Pharmacological stimulation of Adra1a receptors counterintuitively increases wakeful cortical synchrony, and astrocyte-specific removal of *Adra1a* enhances total and arousal-driven neuronal activity, while impairing arousal-related cortical synchrony. Our results directly link astrocytic NE receptors to cortical state regulation. We thus identify NE signaling to astrocytes as a new circuit mechanism by which astrocytes act as sensors of NE changes and synchronize the cortex in response to arousal.

## Results

### Astrocyte Ca^2+^ correlates with increases in pupil diameter

**Fig. 1:**
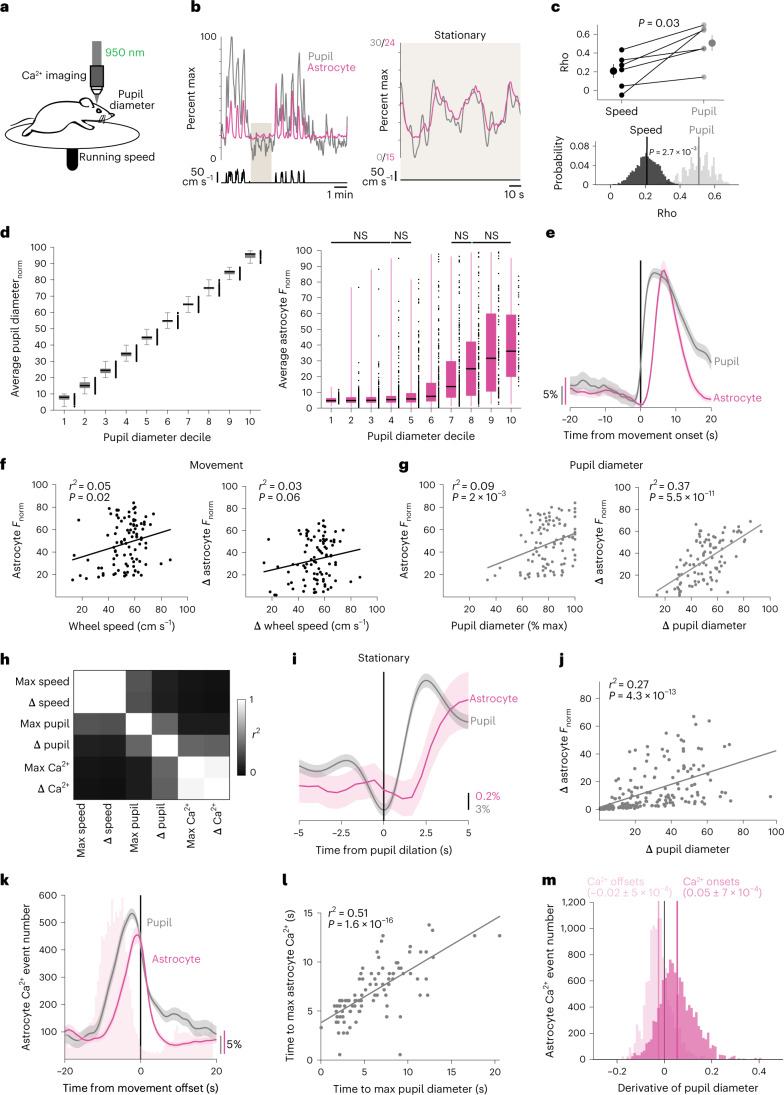
Changes in arousal shape astrocyte Ca^2+^ activity independent of movement. **a**, Experimental setup. **b**, Representative astrocyte Ca^2+^ (magenta), pupil diameter (gray) and wheel speed (black) show that these measures are closely related (left), even during stationary periods (right, smoothed with a five-frame window). Percent max indicates percent of maximum recorded value. **c**, Astrocyte Ca^2+^ correlates better with pupil diameter than speed when comparing across mice (top, two-sided signed-rank test) or using HB of recordings (*n* = 6 mice). Values of *n* are applied throughout the figure. **d**, Left: separating pupil diameter on the basis of size. Right: astrocyte Ca^2+^ was not different within low or high pupil sizes (*n* = 3,206 time bins, one-sided Kruskal–Wallis test, *P* > 0.05 indicated as NS, Supplementary Table 1). Box plot shows median and interquartile range (IQR). Whiskers extend to the most extreme data points. **e**, Astrocyte Ca^2+^ and pupil diameter dynamics aligned to mouse movement onset at *t* = 0 (*n* = 104 movement onsets). **f**,**g**, Linear regression (trend lines) of astrocyte Ca^2+^ and either wheel speed or pupil diameter, after movement onset (*n* = 104 movement onsets, two-sided *t*-test): neither maximum wheel speed (left) nor changes in wheel speed (right) correlate well with astrocyte Ca^2+^ responses (**f**); maximum pupil diameter (left) predicts astrocyte Ca^2+^ less strongly than changes in pupil diameter (right) (**g**). **h**, Heat map of *r*^2^ values between the variables in **f** and **g**. **i**, Astrocyte Ca^2+^ response to pupil dilation during stationary periods (*n* = 188 stationary dilations). **j**, Changes in stationary pupil diameter correlate with astrocyte Ca^2+^ (*n* = 188 stationary dilations, two-sided *t*-test). **k**, Astrocyte Ca^2+^ events (light-pink bars) begin before movement offset, but average astrocyte Ca^2+^ fluorescence (magenta trace) peaks with pupil diameter at the end of movement (*n* = 104 movement offsets). **l**, The latency to maximum astrocyte Ca^2+^ and pupil diameter after movement onset (*n* = 104 movement bouts, two-sided *t*-test) are correlated. **m**, Astrocyte Ca^2+^ events (*n* = 1.17 × 10^4^ astrocyte Ca^2+^ events) begin (dark pink) with pupil dilation (that is, when pupil derivative is positive) and end (light pink) with constriction (negative pupil derivative). Data are presented as mean ± s.e.m. unless otherwise noted.

**Extended Data Fig. 1:**
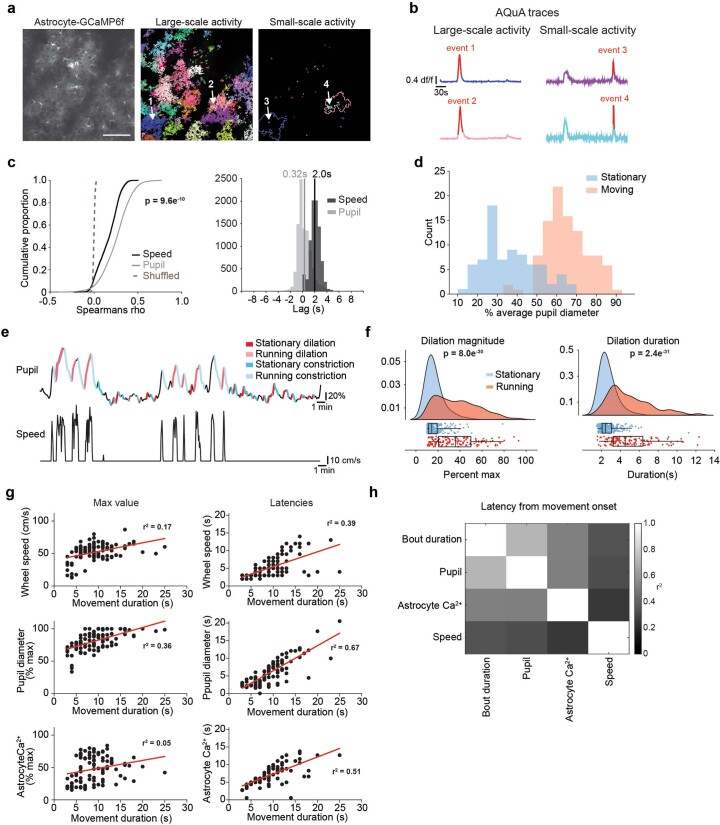
Dissection of astrocyte Ca^2+^ and behavioral state. **(a)** Representative 2P mean projection image from one ten-minute recording of *in vivo* astrocyte GcaMP6f (left, scale bar = 100 µm). AQuA detected both large (middle) and small (right) astrocyte Ca^2+^ events within the entire movie, even when they were spatially overlapping. Arrows indicate two pairs of spatially overlapping events (arrows 1 and 3, and arrows 2 and 4).**(b)** Traces from the AQuA events shown in (a), with the time period of the AQuA-detected event highlighted in red. **(c)** Related to Fig. 1c: Individual astrocyte Ca^2+^ events correlated better with pupil diameter (left, n = 1.2e^4^ Ca^2+^ events, One-sided Kruskal-Wallis test) and had a shorter lag with pupil diameter than wheel speed (right, pupil n = 9.6e^3^, wheel n = 8.3e^3^, rank-sum test). **(d)** Related to Fig. 1d: average pupil diameter during movement (n = 100) and stationary periods (n = 76). **(e)** Classification of behavioral state by both pupil diameter and movement. **(f)** Pupil dilation is smaller (left) and shorter (right, rank-sum tests) during stationary periods (n = 261 dilations, blue) compared with movement-associated dilations (n = 136 dilations, red, boxplots show median and IQR with whiskers to 1.5 * IQR, two-sided Rank Sum test). **(g)** Related to Fig. 1l: Left: Movement duration (n = 104) was related to the maximum wheel speed (top) and pupil diameter (middle), but not to the maximum astrocyte Ca^2+^ (bottom). Right: the latency to the maximum wheel speed (top), pupil diameter (middle), and astrocyte Ca^2+^ (bottom) were strongly linked to movement duration. **(h)** Heatmap summary of the r^2^ between the latencies in (g) right, and movement bout duration.

To first determine whether astrocyte Ca^2+^ activity is dynamically modulated by arousal, we carried out in vivo, 2P Ca^2+^ imaging in visual cortex of awake, head-fixed mice while simultaneously recording pupil diameter and running speed (Fig. 1a). We expressed the Ca^2+^ indicator GCaMP in cortical astrocytes under the GfaABC_1_D promoter and used the Astrocyte Quantitative Analysis (AQuA) toolkit^22^ to accurately capture dynamic fluorescent astrocyte signals, even from spatially overlapping events (Extended Data Fig. 1a,b).

We extracted fluorescence traces from all AQuA-detected Ca^2+^ events and found that movement was accompanied by large increases in astrocyte Ca^2+^—as well as increases in arousal indicated by pupil dilation—consistent with previous reports^6,14,16,23,24^ (Fig. 1b, left). Even during stationary periods, astrocyte Ca^2+^ activity was accompanied by concurrent and proportional fluctuations in arousal (Fig. 1b, right). Population astrocyte Ca^2+^ activity was better correlated with pupil diameter (0.51 ± 0.09) than running speed (0.21 ± 0.07) when comparing across mice (Fig. 1c, top, *n* = 6 mice) or using hierarchical bootstrapping (HB, Fig. 1c, bottom). In addition, individual astrocyte Ca^2+^ events correlated better with arousal compared with either speed- or time-shuffled data (Extended Data Fig. 1c, left). Most Ca^2+^ events (9,597/11,674; 82.2%) exhibited a maximal cross-correlation with pupil diameter within 10 s and a short (0.32 ± 0.01 s standard error of the mean (s.e.m)) lag, consistent with arousal driving astrocyte activity (Extended Data Fig. 1c, right).

Our analysis indicates that arousal contributes to astrocyte Ca^2+^ beyond its association with movement. However, cortical activity reflects both absolute pupil diameter^23^ and relative changes in pupil diameter^24^. To investigate whether astrocytes sense absolute levels of arousal, we binned the pupil diameter into deciles (Fig. 1d, left), and calculated the average Ca^2+^ fluorescence in each. Astrocyte Ca^2+^ dynamically varied with pupil diameter only when the pupil was ~40–80% of maximum diameter (Fig. 1d, right, and Supplementary Table 1). This range matched the overlap in pupil diameter found between stationary and movement periods (Extended Data Fig. 1d), indicating this relationship was a function of movement, and suggesting that absolute pupil diameter did not adequately explain the relationship between arousal and astrocyte Ca^2+^.

To test whether relative changes in pupil diameter were linked to astrocyte Ca^2+^ responses, we calculated an event-triggered average relative to the start of movement. As expected, we found that pupil diameter and—with a short delay—astrocyte Ca^2+^ increased around movement onset (Fig. 1e). We then asked which behavioral variables best explained movement-evoked astrocyte Ca^2+^ activity (Fig. 1f–h). We found that speed was a poor predictor of the maximal astrocyte Ca^2+^ fluorescence (Fig. 1f, left) as previously reported^16^, as was the relative change in speed (Fig. 1f, right). In addition, absolute pupil diameter following movement had a weak correlation with astrocyte Ca^2^ fluorescence, indicating that, even during behavioral state changes, absolute pupil diameter is a poor predictor of astrocyte responses (Fig. 1g, left). In contrast, the relative change in pupil diameter explained a substantial portion of the astrocyte Ca^2+^ response to movement, indicating that astrocytes are specifically sensitive to relative changes in arousal (Fig. 1g, right).

We further dissociated the effects of movement and arousal on astrocytes by separating behavioral state periods (Extended Data Fig. 1e). The relationship between astrocyte Ca^2+^ and increases in pupil diameter persisted during stationary periods (Fig. 1i–j), even though pupil diameter changes were smaller (Extended Data Fig. 1f). These results demonstrate that astrocytes are sensitive to smaller changes in arousal than previously recognized.

We also wondered whether changes in arousal could explain the timing of astrocyte Ca^2+^ activity. We noticed that astrocyte Ca^2+^ peaked with pupil diameter around movement offset (Fig. 1k) and the time to maximum pupil diameter was strongly correlated with the time to peak astrocyte activity (Fig. 1l). Both were also dependent on the duration of the movement bout (Extended Data Fig. 1g). When examining all astrocyte Ca^2+^ events, onsets occurred more often during dilation and offsets during constriction (Fig. 1m). These findings indicate that changes in arousal shape the timing and level of astrocyte Ca^2+^ activity.

### Phasic increases in NE precede astrocyte Ca^2+^

**Fig. 2:**
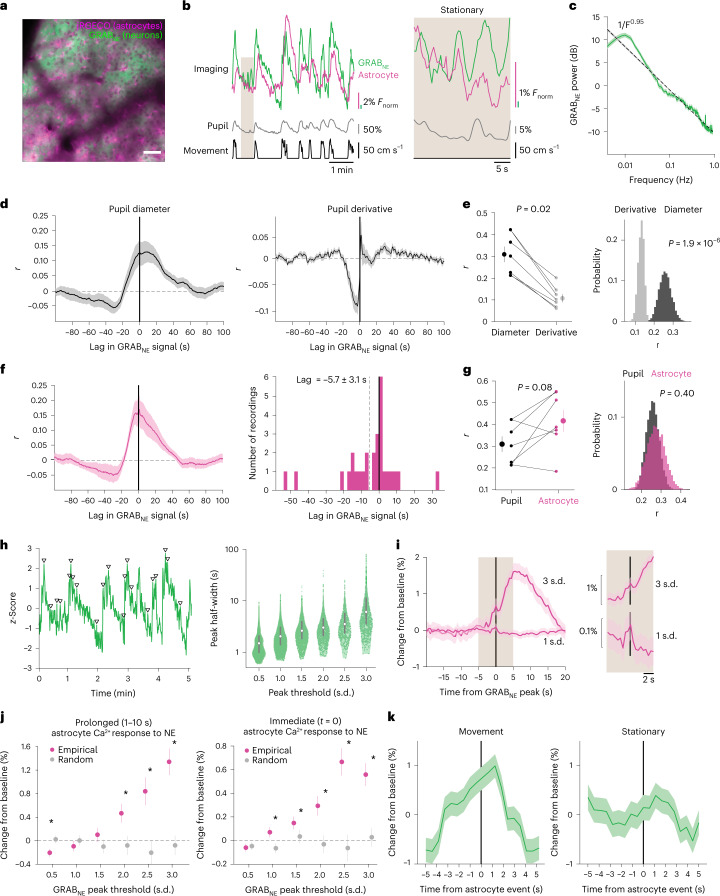
Astrocytes are sensitive to a range of NE increases. **a**, In vivo 2P image showing dual-color expression of neuronal GRAB_NE_ and astrocyte jRGECO1b. Scale bar, 50 µm. *n* = 28 recordings from seven mice throughout figure. **b**, Representative traces smoothed with a five-frame window. Astrocyte Ca^2+^ (magenta), GRAB_NE_ (green), pupil diameter (gray) and wheel speed (black) show a close relationship over the course of minutes (left), and over the course of seconds during stationary periods (right). **c**, The power spectrum of GRAB_NE_ dynamics shows an inverse relationship with frequency (*F*^−0.95^, dotted line) and increased power in slow fluctuations (period >30 s, mean with jackknifed error bars). **d**, GRAB_NE_ positively correlates with pupil diameter (left), but not the derivative of pupil diameter (right). **e**, GRAB_NE_ is more strongly correlated with pupil diameter than pupil derivative across mice (left, two-sided signed-rank test) and across recordings (HB). **f**, Left: astrocyte Ca^2+^ activity positively correlates with GRAB_NE_ activity. Right: astrocyte Ca^2+^ activity follows changes in GRAB_NE_. **g**, No difference was found between the GRAB_NE_ correlation with astrocytes compared with pupil diameter across mice (left, *P* = 0.08, two-sided signed-rank test) or across recordings (HB). **h**, Left: phasic increases in GRAB_NE_ signal, with arrowheads marking a subset of peaks. Right: larger increases in GRAB_NE_ had longer durations (*P* < 0.05 for all bins, one-sided Kruskal–Wallis test, *n* and *P* values listed in Supplementary Table 2). Box plot shows median and IQR. **i**, Example astrocyte Ca^2+^ traces separated by GRAB_NE_ peak amplitude. Left: astrocyte Ca^2+^ activity showed large and persistent responses to large increases (for example, 3 s.d.) in GRAB_NE_. Right (shaded area, 5 s before and after *t* = 0): both large and small (for example, 1 s.d.) changes in GRAB_NE_ drove small transient increases in astrocyte Ca^2+^. **j**, Left: astrocyte Ca^2+^ persistently increased after large (≥2 s.d.) increases in GRAB_NE_, and scaled with GRAB_NE_ amplitude (*P < 0.05, two-sided rank-sum test, *n* and *P* values listed in Supplementary Table 3). Right: astrocyte Ca^2+^ showed proportional, time-locked responses to even small changes (≥1 s.d.) in GRAB_NE_ (*P < 0.05, two-sided rank-sum test; for details, see Supplementary Table 3). **k**, GRAB_NE_ activity around astrocyte Ca^2+^ onsets at *t* = 0 s (*n* = 1.2 × 10^4^ events) shows increased extracellular NE before astrocyte Ca^2+^ events in both movement and stationary periods. Data are presented as mean ± s.e.m.

**Extended Data Fig. 2:**
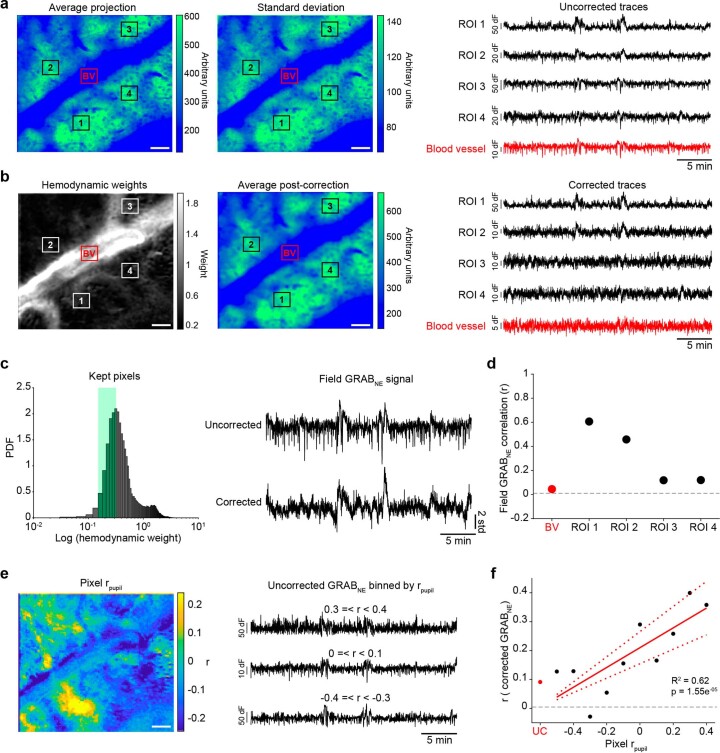
Hemodynamic correction of 2P GRAB_NE_ signals. **(a)** Left: Average projection of a single GRAB_NE_ recording with four regions-of-interest (ROI) in areas with clear GRAB_NE_ fluorescence (black squares, labeled 1–4) and one ROI in a blood vessel (red square, labeled BV). Middle: standard deviation projection. Right: average fluorescence in each ROI before hemodynamic correction. **(b)** Left: Estimated hemodynamic signal present in each pixel. Middle: recovered average projection after hemodynamic correction. Note average fluorescence after hemodynamic correction is broadly similar the uncorrected data in (a) with some blurring due to Gaussian smoothing in preprocessing. Right: average ROI fluorescence after hemodynamic correction. **(c)** ROI-free methodology for obtaining GRAB_NE_ signal. Left: Following hemodynamic correction, the bottom quartile of pixels with the lowest hemodynamic weights, excluding the bottom 1% which often had artefactual signals, were kept. Right: These pixels were averaged together to produce the corrected GRAB_NE_ signal (bottom) which had substantially less hemodynamic contamination than the uncorrected field fluorescence (top). **(d)** Correlation between the corrected GRAB_NE_ signal and the uncorrected ROIs in (a) and (b). **(e)** Left: Correlation between uncorrected GRAB_NE_ fluorescence and pupil diameter. Right: Example traces from the average of pixels that were positively correlated (top), negatively correlated (bottom), or showed little relationship (middle) with pupil diameter. **(f)** The corrected GRAB_NE_ signal is not similar to the uncorrected GRAB_NE_ signal (red dot) but instead reflects the GRAB_NE_ signal of binned pixels (black dots) correlated to pupil diameter (R^2^ = 0.62, p = 1.55^−5^, two-sided t-test).

NE is a key driver of both changes in pupil diameter and brain activity^25^. While recent work suggests that astrocytes preferentially respond via Ca^2+^ to “multi-peaked” NE axonal activity^15^, the impact of behavioral state has not yet been considered. To determine the relationship between NE and astrocyte Ca^2+^ activity, we simultaneously expressed a fluorescent sensor of NE, GRAB_NE_^26^, in cortical neurons under the h-syn promoter and the Ca^2+^ indicator jRGECO1b in cortical astrocytes under the GfaABC_1_D promoter (Fig. 2a and Supplementary Video 1). Examining the raw GRAB_NE_ fluorescence, we saw reductions in fluorescence that matched changes in background vasculature, independent of indicator expression (Extended Data Fig. 2a). Our 2P imaging paradigm was not compatible with techniques to directly measure hemodynamic effects such as reflectance imaging and blind-source separation^27^. Therefore, we developed a method to approximate and compensate for hemodynamic signals in 2P imaging (Extended Data Fig. 2b and Methods). To further limit hemodynamic contamination, we took the mean of the corrected GRAB_NE_ signal, excluding highly contaminated or artifactual pixels (Extended Data Fig. 2c). As our methodology only approximates hemodynamic artifacts and does not account for possible hemodynamics effects from excitation or out-of-plane GRAB_NE_ fluorescence, we next sought to confirm the accuracy of our methodology. We found our corrected GRAB_NE_ signal showed little correlation with vasculature or hemodynamically contaminated regions (*r* < 0.2) but correlated well with regions that had active GRAB_NE_ signal (Extended Data Fig. 2d). To further validate our methodology in an unbiased manner, we correlated individual pixels with pupil diameter (Extended Data Fig. 2e) and found the corrected GRAB_NE_ signal linearly correlated with pupil diameter-related pixels suggesting our method reflects GRAB_NE_ signal that occurs from pupil-related increases in NE while reducing hemodynamic effects (Extended Data Fig. 2f).

Using our corrected GRAB_NE_ signal, we found that GRAB_NE_ fluorescence matched changes in pupil diameter and astrocyte Ca^2+^ activity, although the GRAB_NE_ showed a slow decay, similar to previous reports^15^ (Fig. 2b, left). This relationship persisted even during stationary periods (Fig. 2b, right). The slow GRAB_NE_ decay was reflected in the power spectrum, which showed an inverse relationship with frequency (Fig. 2c). Slow (0.01–0.03 Hz) GRAB_NE_ fluctuations had more power than the empirically fit 1/*f* relationship, suggesting long-lasting fluctuations in “tonic” NE predominate the GRAB_NE_ signal.

To confirm the link between GRAB_NE_ and arousal, we examined the cross-correlation between pupil diameter and GRAB_NE_. We found a positive correlation between GRAB_NE_ and spontaneous changes in pupil diameter (*r* = 0.13, Fig. 2d), similar to that previously described for cortical NE axons^28^. However, the maximum cross-correlation was broad and continued for at least 20 s after the pupil diameter (Fig. 2d, left). Further, unlike what has been reported for NE axons^28^, GRAB_NE_ showed poor cross-correlation (*r* = 0.04) with the derivative of pupil diameter (Fig. 2d right, and 2e). These results suggest that cortical GRAB_NE_ dynamics best report arousal level rather than changes in arousal, and may substantively differ from the activity pattern of the underlying NE axons.

We next quantified the relationship between GRAB_NE_ and astrocyte Ca^2+^ activity. We found a positive, broad correlation (*r* = 0.17) between the two (Fig. 2f, left), with GRAB_NE_ preceding astrocyte Ca^2+^ (−5.7 ± 3.1 s delay, Fig. 2f, right) and GRAB_NE_ showing a similar link to astrocyte Ca^2+^ as arousal (Fig. 2g). Although slow NE fluctuations predominated our GRAB_NE_ signal (Fig. 2c), we also noticed phasic increases in NE (Fig. 2h, left, triangles). We segregated phasic NE peaks by amplitude (Fig. 2h, left, triangles) and saw that larger phasic GRAB_NE_ activity was also longer lasting (Fig. 2h, right, and Supplementary Table 2). We then examined how astrocyte Ca^2+^ changed following phasic NE activity. We saw that large (≥2 standard deviations (s.d.)) phasic changes in NE preceded prolonged increases in astrocyte Ca^2+^ (Fig. 2i–j, left, and Supplementary Table 3, left), while small increases in GRAB_NE_ co-occurred with proportional increases in astrocyte Ca^2+^ (Fig. 2i, right). These increases were observed even with smaller (1 s.d.) changes in NE (Fig. 2j, right, and Supplementary Table 3, right). These results suggest astrocytes dynamically respond to phasic changes in NE, in both the duration and amplitude of their Ca^2+^ signaling. We also calculated the event-triggered average GRAB_NE_ signal relative to astrocyte Ca^2+^ events and confirmed that GRAB_NE_ increased before astrocyte Ca^2+^, and peaked shortly after astrocyte Ca^2+^ signaling had begun (Fig. 2k). This relationship was true for both astrocyte Ca^2+^ events during movement (Fig. 2k, left) and stationary periods (Fig. 2k, right). In sum, these results indicate that astrocytes are sensitive to changes in NE across behavioral states.

**Extended Data Fig. 3:**
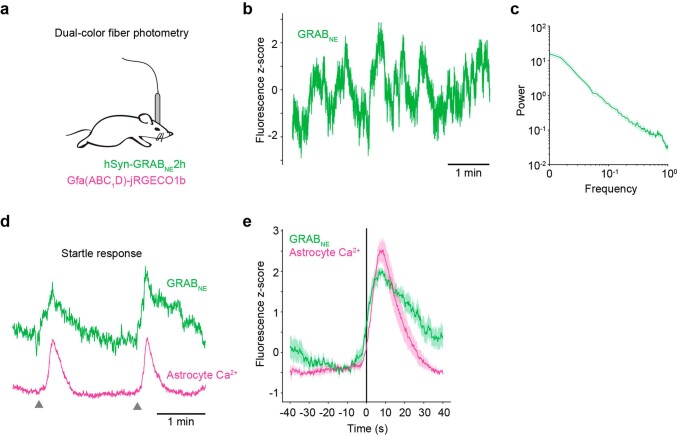
Freely moving fiber photometry recordings of GRAB_NE_ and astrocyte Ca^2+^. **(a)** Schematic of fiber photometry recording set-up. **(b)** A 5-minute example fiber photometry recording of GRAB_NE_ signal. **(c)** GRAB_NE_ power spectrum. n = 4 mice, 19 recordings over multiple days, 10–30 minutes/recording. Error lines are ± s.e.m. across all mice.) **(d)** GRAB_NE_ (green) and astrocyte jRGECO1b (magenta) signals evoked by startle responses due to tail lifts (grey arrow heads). **(e)** Average startle-response evoked traces for GRAB_NE_ and jRGECO, aligned to onset of astrocyte Ca^2+^. n = 4 mice, 4 tail lifts per mouse. Error is ± s.e.m. across mice.

To corroborate these findings, we performed freely moving, dual-color fiber photometry recordings of GRAB_NE_ and astrocyte Ca^2+^ (Extended Data Fig. 3a) and saw similar GRAB_NE_ dynamics (Extended Data Fig. 3b,c). To confirm the relationship between NE and astrocyte Ca^2+^, we used tail lifts to evoke startle responses^14^, and saw expected increases in both GRAB_NE_ and astrocyte Ca^2+^ (Extended Data Fig. 3d). The GRAB_NE_ signal began increasing before astrocyte Ca^2+^ and persisted after the startle response (Extended Data Fig. 3e). These results further support the hypothesis that astrocytes respond to phasic increases in NE.

### Astrocyte Ca^2+^ may reduce arousal-driven neuronal activity

**Fig. 3:**
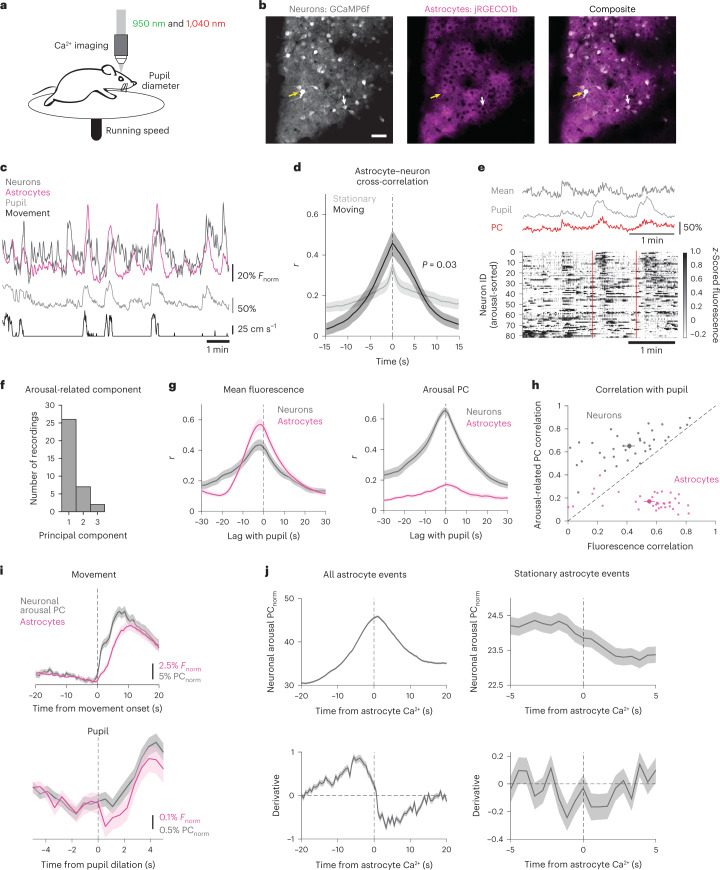
Astrocyte Ca^2+^ is positioned to reduce effects of arousal on population-level neuronal activity. **a**, Experimental paradigm for dual-color Ca^2+^ imaging of neurons and astrocytes. *n* = 33 recordings from eight mice throughout figure. **b**, 2P images from one recording of in vivo neuronal GCaMP6f (gray) and astrocyte jRGECO1b (magenta). Yellow arrows indicate bleed-through in the red channel which was accounted for (Methods), while white arrows show a neuron with no bleed through. Scale bar, 50 µm. **c**, Example of neuronal (dark gray) and astrocyte (magenta) Ca^2+^ activity, with pupil diameter (light gray) and wheel speed (black). **d**, Astrocyte and neuronal Ca^2+^ were positively correlated, and increased their correlation during movement compared with stationary periods (two-sided rank sum test). **e**, Top: an example of pupil diameter (light gray) fluctuations alongside changes in the neuronal mean fluorescence (dark gray) and arousal-associated PC of neuronal activity (red). Bottom: an example heat map of single-cell neuronal activity sorted by arousal PC weight shows that the arousal PC captured heterogeneous neuronal responses to arousal, including around movement events (red dashed lines). **f**, The component number of the arousal PC. **g**, Both neurons (gray) and astrocytes (pink) showed positive correlations between mean Ca^2+^ fluorescence and pupil diameter (left). Using PC analysis, we identified neuronal activity that showed strong correlation with pupil diameter, whereas astrocyte Ca^2+^ activity was not amenable to this analysis. **h**, Scatter plot of the correlation between either arousal PC (*y* axis) or mean fluorescence (*x* axis) for each recording. PC analysis identified arousal-associated neuronal activity for neurons (gray dots), but not astrocytes (magenta dots). **i**, Astrocyte Ca^2^ signaling (magenta) occurred alongside increases in arousal-associated neuronal activity (gray), and peaked with reductions in neuronal activity, for both movement (top, *n* = 134 movement bouts) and stationary increases in arousal (bottom, *n* = 772 pupil dilations). **j**, Top: arousal-associated neuronal activity tends to peak and then decrease around astrocyte Ca^2+^ activity both overall (left, *n* = 8.9e^4^ events) and during stationary periods (right, *n* = 3.2 × 10^4^ events). Bottom: the derivative of arousal-associated neuronal activity demonstrates that arousal-associated neuronal activity decreases directly following astrocyte Ca^2+^ events both overall (left) and during stationary periods (right). All data are presented as mean ± s.e.m. unless otherwise noted.

We next wondered how arousal-mediated changes in nearby neurons relate to astrocyte Ca^2+^ activity, as arousal also strongly modulates neuronal activity^6,24,29^. To answer this, we expressed hSyn-GCaMP6f in neurons and GfaABC_1_D-jRGECO1b in astrocytes to record the Ca^2+^ activity of both cellular populations simultaneously (Fig. 3a,b and Supplementary Video 2).

We found that astrocyte and neuronal Ca^2+^ fluctuated together along with movement and pupil diameter (Fig. 3c). The average fluorescence of neurons and astrocytes were maximally correlated with no time shift, a relationship enhanced during movement (Fig. 3d, black, *r* = 0.46 ± 0.06) compared with stationary periods (Fig. 3d, gray, *r* = 0.34 ± 0.04). We next investigated how astrocyte Ca^2+^ relates to arousal-driven neuronal activity specifically. To do this, we used principal component (PC) analysis to identify the PC of neuronal activity most correlated with arousal (arousal PC)^29^. We found that the arousal PC of neuronal activity (Fig. 3e, top, red line) often better matched the pupil diameter (Fig. 3e, top, light gray) than the mean neuronal activity (Fig. 3e, top, dark gray), probably due to the variability in neuronal responses to arousal (Fig. 3e, bottom).

In agreement with previous work^29^, we found that the arousal PC was usually PC1 (Fig. 3f, gray, 28/35 recordings). Furthermore, we found that average astrocyte Ca^2+^ activity (Fig. 3g, left, magenta) was better correlated with pupil diameter than average neuronal Ca^2+^ activity (Fig. 3f, left, gray). This relationship was reversed when using the arousal PC of astrocyte (Fig. 3g, right, magenta) and neuronal (Fig. 3g, right, gray) activity instead. The arousal PC consistently reflected arousal-associated activity better than using mean fluorescence for neurons (Fig. 3h, gray dots) but not for astrocytes (Fig. 3h, magenta dots), suggesting that astrocytic responses to arousal are best characterized by a consistent increase reflected in the mean fluorescence while neuronal responses are best captured by describing the variability in neuronal activity using PC analysis.

We next looked at changes in astrocyte Ca^2+^ and arousal-driven neuronal activity with arousal. We found that, with both movement (Fig. 3i, top) and stationary increases in pupil diameter (Fig. 3i, bottom), arousal-associated neuronal activity (Fig. 3i, gray) increased alongside astrocyte Ca^2+^ (Fig. 3i, magenta) and astrocyte Ca^2+^ appeared to peak when neuronal activity began to diminish (Fig. 3i). To investigate this further, we looked at the average arousal PC around the onset of all astrocyte Ca^2+^ events. We similarly found that astrocyte Ca^2+^ events occurred at the peak of arousal-associated neuronal activity (Fig. 3j, top left), and using the first derivative of the neuronal PC (Fig. 3j, bottom left), we saw astrocyte Ca^2+^ occurred at a transition point between increasing and decreasing arousal-associated neuronal activity. This was also true for astrocyte Ca^2+^ events during stationary periods (Fig. 3j, right). These results indicate that astrocyte activity is positioned to counteract the effects of arousal on the local neuronal population.

**Extended Data Fig. 4:**
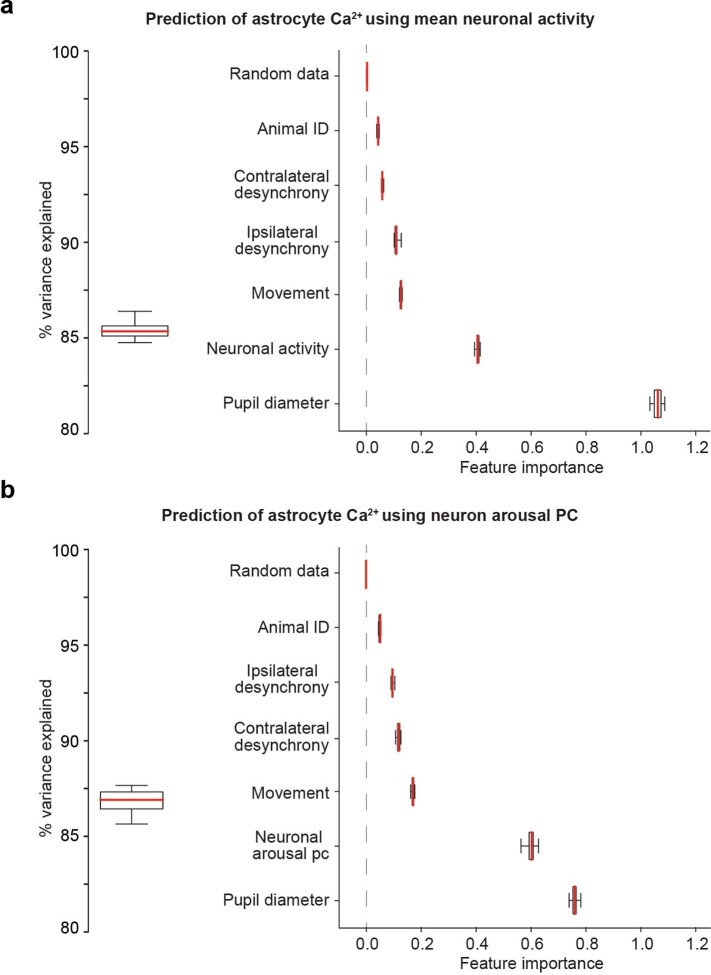
Analysis of contributions to astrocyte Ca^2+^. **(a)** Left: A Random Forest Regression model was trained (80% of data, 2.3e^4^ samples) to predict (20% of data, 5.6^3^ samples) average astrocyte Ca^2+^ fluorescence accurately (mean r^2^ = 0.85 ± 4.5e^-3^ std, n = 10 cross-validations). Right: Relative feature importance based on permutation testing of each predictor. Randomly generated data was included as a negative control and did not inform model predictions. **(b)** Same analysis as in (a), using the neuronal arousal PC rather than average neuronal activity. Random Forest Regression using the neuronal arousal PC showed a similar accuracy to that using mean neuronal fluorescence (mean r^2^ = 0.87 ± 6.4e^-3^ std, n = 10 cross-validations). Boxplots show median and IQR with whiskers to 1.5 * IQR.

These results raised the possibility that astrocyte Ca^2+^ may respond to changes in local arousal-associated neuronal activity, rather than arousal or NE per se. To dissect the effects of neuronal activity and arousal on astrocyte Ca^2+^ we used Random Forest Regression to predict astrocyte Ca^2+^ (Extended Data Fig. 4). Using this strategy, we could explain ~85% of the variance in astrocyte Ca^2+^ fluorescence (Extended Data Fig. 4a, left). Permutation-based feature importance analysis indicated that pupil diameter was the most important predictor of astrocyte Ca^2+^, and a substantially better predictor than the next most important factor, local neuronal Ca^2+^ fluorescence (Extended Data Fig. 4a, right). These results remained consistent when using the neuronal arousal PC data (Extended Data Fig. 4b). We interpret these results as evidence that astrocytes, while responsive to local neuronal activity, are sensitive to arousal beyond the influence of nearby neurons.

### Arousal-driven astrocyte Ca^2+^ is not local neuron-dependent

**Fig. 4:**
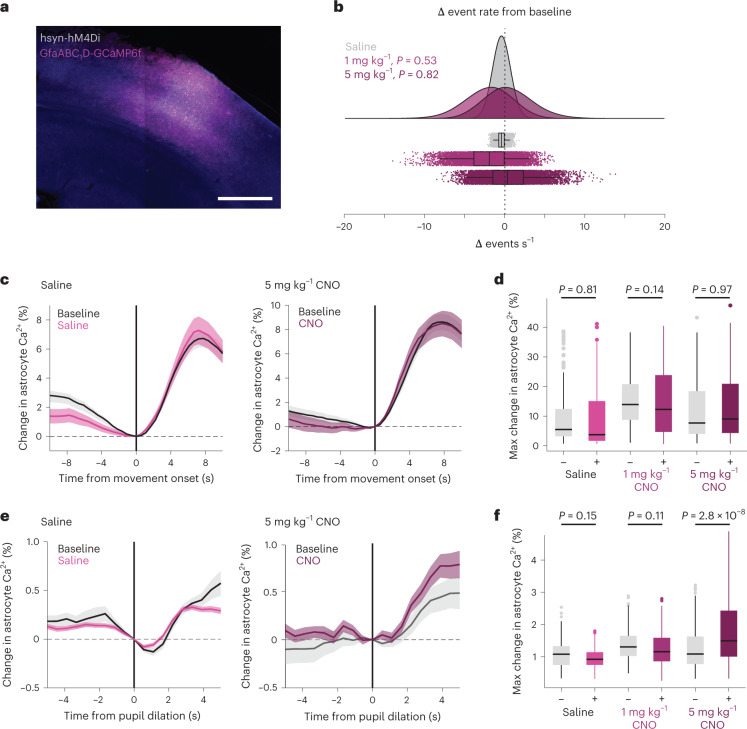
Arousal-driven astrocyte Ca^2+^ is not dependent on local neuronal activity. **a**, Confocal image showing representative cortical expression from one mouse of both astrocyte GCaMP6f (magenta) and neuronal inhibitory DREADD hM4Di (gray). Scale bar, 500 µm. **b**, Bootstrapped change in astrocyte event rate (*n* = 1 × 10^5^ resampled events from five mice) after saline (gray) or CNO (purple) administration. No significant change in astrocyte Ca^2+^ event rate was found (*P* < 0.05, One-sided test of the proportion of bootstrapped change in event rates below saline). **c**, Average change in astrocyte Ca^2+^ response to movement after saline (left) or CNO (right, 5 mg kg^−1^) compared with the baseline in each animal (black lines). **d**, No significant (*P* < 0.05) difference in astrocyte Ca^2+^ responses (% change in astrocyte Ca^2+^ relative to *t* = 0; box-and-whisker plot with outliers as open circles), to movement after saline or either 1 mg kg^−1^ or 5 mg kg^−1^ of CNO was found (one-sided Kruskal–Wallis test, *n* and *P* values listed in Supplementary Table 4). **e**, Average change in astrocyte Ca^2+^ response to stationary pupil dilation after saline (left) or CNO (right, 5 mg kg^−1^). **f**, No significant difference (*P* < 0.05) was found within saline or 1 mg kg^−1^ CNO conditions. 5 mg kg^−1^ CNO caused an enhancement in astrocyte responses to arousal (Kruskal–Wallis test, *n* and *P* values listed in Supplementary Table 5). Box plots show median and IQR, with whiskers extended to 1.5× IQR. Line plots are presented as mean ± s.e.m.

To further investigate how arousal and local neuronal activity drive arousal-associated astrocyte Ca^2+^, we expressed hM4Di, an inhibitory Gi-coupled Designer Receptor Exclusively Activated by Designer Drugs (DREADD)^30^ in neurons, as well as GCaMP6f in astrocytes to image Ca^2+^ (Fig. 4a). We found no difference in the average frequency of astrocyte Ca^2+^ events from baseline following administration of either the hM4Di agonist CNO or saline, although the overall variability increased (Fig. 4b, resampled distributions from each condition). We also found no difference in astrocyte Ca^2+^ responses to movement with either saline or CNO administration (Fig. 4c,d and Supplementary Table 4). There was no difference in astrocyte Ca^2+^ responses to arousal during stationary periods, except at a high CNO concentration (5 mg kg^−1^), where we recorded a potential enhancement of arousal-associated Ca^2+^ (Fig. 4e,f and Supplementary Table 5). These results indicate that local neuronal activity is not necessary for astrocyte Ca^2+^ responses to arousal, and that astrocytes may monitor the state of local circuit activity when responding to arousal.

### NE ties astrocyte Ca^2+^ to changes in cortical state

**Fig. 5:**
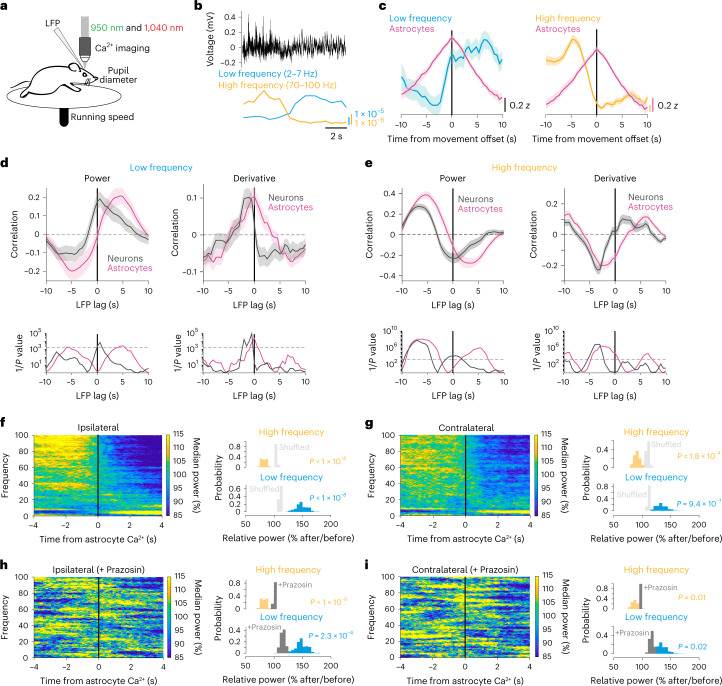
NE-dependent astrocyte Ca^2+^ occurs at the crux of cortical state changes. **a**, Experimental setup. **b**, Example LFP data (black) with calculated LF (2–7 Hz, blue) and HF (70–100 Hz, orange) power. **c**, Average LFP LF (left) and HF (right) power with astrocyte Ca^2+^ activity (magenta) around movement offset at *t* = 0 s (*n* = 52 offsets from four mice). **d**, Top: cross-correlation between astrocyte (magenta) or neuronal Ca^2+^ (gray) activity and LF power (left), or the derivative of LF power (right). Bottom: significance (1/*P*, two-sided signed-rank test) for astrocytes and neurons. Dashed line is the threshold of correction for multiple comparison. **e**, HF power and its derivative are cross-correlated to neuronal and astrocyte Ca^2+^, as for LF power in **d**. Neuronal Ca^2+^ correlated with LFP power, while astrocyte Ca^2+^ correlated with the derivative of LFP power. **f**,**g**, LFP power dynamics in ipsilateral (**f**) and contralateral (**g**) cortex, centered around astrocyte Ca^2+^ onsets at *t* = 0 s. LFP power in a 5 s window around astrocyte Ca^2+^ events was computed, and each frequency was normalized by its median power. (All p-values from one-sided HB). Left: astrocyte Ca^2+^ events occur at the crux of ipsilateral (**f**, *n* = 4 mice) and contralateral (**g**, *n* = 5 mice) LFP state transitions from HF- to LF-dominated states. Comparisons are made using HB between empirical data (colored bars) and shuffled distributions (light gray). Right: HF power decreased (orange) and LF power (blue) increased after astrocyte Ca^2+^ events. **h**,**i**, This relationship was abolished in both ipsilateral (**h**) and contralateral (**i**) cortical LFP recordings after administration of the Adra1 receptor antagonist Prazosin (5 mg kg^−1^, i.p., *n* = 4 mice), for both LF and HF power (comparisons are made using HB between state changes without Prazosin (colored bars) and with the addition of Prazosin (dark gray). Line plots are presented as mean ± s.e.m.

In Fig. 3, we showed that astrocyte Ca^2+^ events occur before reductions in arousal-associated neuronal activity. We wondered whether astrocyte Ca^2+^ was similarly related to activity changes in the broader cortical circuit. To answer this question, in a subset of mice expressing Ca^2+^ indicators in neurons (hysn-GCaMP6f) and astrocytes (GFAP-jRGECO1b), we implanted low-impedance electrodes bilaterally into the visual cortex (Fig. 5a). Using this methodology, we recorded the LFP (Fig. 5b, top), which unlike our spatially restricted imaging window, reflects the integrated electrical inputs and activity of the visual cortex and surrounding brain regions^31^. In these LFP recordings, we observed spontaneous transitions between cortical states dominated by LF power (2–7 Hz; running-evoked 7–10 Hz was excluded) and those dominated by HF (70–100 Hz) power, indicative of synchronous and desynchronized cortical states respectively (Fig. 5b)^1^. We noticed that, during transitions to a low-arousal state following movement, astrocyte Ca^2+^ peaked as LF power increased (Fig. 5c, left) and HF power decreased (Fig. 5c, right).

To investigate this relationship, we cross-correlated astrocyte and neuronal Ca^2+^ with LFP power, and with the derivative of LFP power. We found that astrocyte cross-correlations with LFP power were right-shifted from the equivalent neuronal cross-correlations (Fig. 5d,e). When looking at LF power specifically (Fig. 5d, left, magenta), we found that astrocyte Ca^2+^ preceded increases in LF power (Fig. 5d, left, magenta). In contrast, neuronal Ca^2+^ was only significantly positively correlated with LF power with zero lag (Fig. 5d, left, gray), indicating that LF power and neuronal population activity are coordinated. These findings were confirmed when examining the derivative of LF power. We found that changes in LF power were maximally correlated with astrocyte activity without any lag (Fig. 5d, right, magenta), while LF power changes preceded neuronal activity (Fig. 5d, right, gray). Our results demonstrate that, while neuronal Ca^2+^ is correlated with LF power, astrocyte Ca^2+^ is correlated with changes in LF power.

We found a similar relationship when using HF power, although inverted about the *y* axis. HF power preceded both neuronal and astrocyte Ca^2+^, with neurons maximally negatively correlated with zero time lag (Fig. 5e, left). In addition, both astrocyte and neuronal Ca^2+^ were negatively correlated with the derivative of HF power, but for astrocytes this relationship was true both when HF power preceded or had no lag with astrocyte Ca^2+^ (Fig. 5e, right). These results indicate that, while HF power precedes both neuronal and astrocyte Ca^2+^, neuronal Ca^2+^ is inversely related to absolute HF power while astrocyte Ca^2+^ is inversely related to changes in HF power. Overall, this analysis demonstrates that neuronal Ca^2+^ activity reflects absolute LFP power while astrocyte Ca^2+^ is instead tied to changes in LFP power.

We next asked whether arousal-driven astrocyte Ca^2+^ was generally tied to changes in LFP power by computing an astrocyte Ca^2+^ event-triggered spectrogram. We found that astrocyte Ca^2+^ signaling occurred at the crux of a transition from a HF-dominated cortical state to one dominated by LF power (Fig. 5f). This relationship was also seen in the LFP power of the contralateral cortex (Fig. 5g), indicating that astrocyte Ca^2+^ is tied to the transition of neuronal activity from a high- to low-arousal state not just at a local level (Fig. 3j), and at the level of the nearby cortex (Fig. 5f), but also to bi-hemispheric changes in cortical state.

On the basis of our identified changes in arousal and NE as major drivers of spontaneous changes in astrocyte Ca^2+^ (Figs. 1 and 2), we hypothesized that NE was necessary for the link between astrocyte Ca^2+^ and cortical state transitions. In particular, we hypothesized that Adra1 receptors might underlie this relationship due to their importance for astrocyte physiology^14,16,19,32^. We pharmacologically blocked Adra1 receptors using Prazosin (5 mg kg^−1^, intraperitoneal (i.p.)) and found that the relationship between astrocytes and cortical state was reduced (Fig. 5h,i), confirming that NE signaling links astrocyte Ca^2+^ to cortical state changes.

### Enrichment of Adra1a mRNA in visual cortex astrocytes

**Extended Data Fig. 5:**
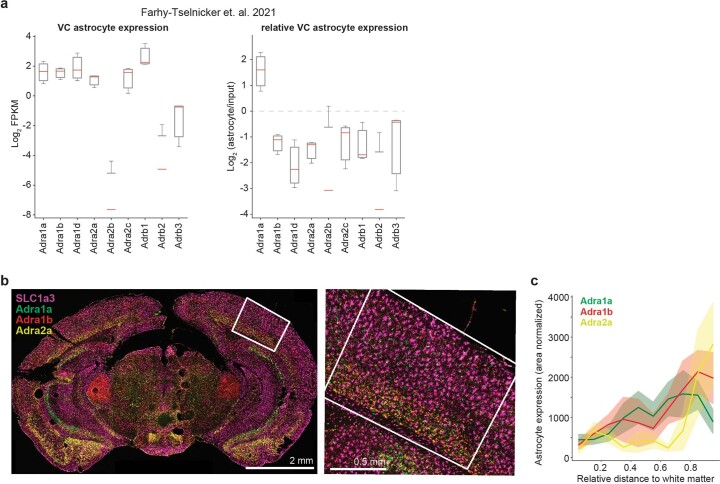
Cortical astrocyte expression of adrenergic receptors. **(a)** Ribosomal-mRNA expression in visual cortex astrocytes of P120 mice from the Farhy-Tselnicker et. al. publicly available dataset. Visual cortex astrocytes show expression of many adrenergic receptors (left) but preferentially express the Adra1a receptor relative to input control (right). Boxplots show median and IQR with whiskers to 1.5 * IQR (N = 3 mice for astrocyte ribosomal-mRNA and 1 input control). **(b)** Left: Representative brain section with Adra1a (green), Adra1b (red), and Adra2a (yellow) mRNA labeled by smFISH using the LaST map pipeline^34^. SLC1a3 (GLAST) mRNA expression (purple) was used to define the location of astrocytes. The white square shows a representative ROI used to quantify visual cortex expression. Right: High-magnification image of visual cortex, showing receptor mRNA both within astrocytes marked by high GLAST mRNA (purple), and outside of astrocytes. **(c)** Quantification of NE-receptor mRNA expression in visual cortex astrocytes, quantified by relative cortical depth. Line plots show mean ± std (n = 3 mice, 7 ROIs).

We next sought to determine which Adra1 receptors were most likely to underlie the connection between astrocytes and cortical state. We analyzed a previously published dataset that profiled the ribosome-associated messenger RNA expression of astrocytes in the mouse visual cortex^33^. We looked at adrenergic receptor expression in the adult (P120) dataset and confirmed astrocytic expression of most adrenergic receptors, although astrocytes showed little Adra2b, Adrab2 and Adrab3 in this dataset (Extended Data Fig. 5a, left). When analyzing the astrocytic expression of adrenergic receptors relative to an input control, the relative expression of Adra1a mRNA was highest and greater than the input control, indicating that Adra1a mRNA may be preferentially enriched in visual cortex astrocytes (Extended Data Fig. 5a, right).

We also used spatial transcriptomics to assess the mRNA expression of Adra1a, Adra1b and Adra2a (Extended Data Fig. 5b). Using LaST map single-molecule in situ hybridization (smFISH)^34^, we saw striking heterogeneity throughout the brain (Extended Data Fig. 5b, left). All three receptors showed cortical expression in astrocytes, as delineated by expression of the astrocyte-specific mRNA SlcA3 (GLAST), and in non-astrocytic cell types (Extended Data Fig. 5b, right). We quantified mRNA spots per astrocyte in the visual cortex for each of these receptors and found higher expression at deep layers of cortex (Extended Data Fig. 5c). This was particularly true for Adra2a and Adra1b receptors, while Adra1a receptors also showed higher expression in intermediate layers of cortex (Extended Data Fig. 5c), where the effects of cortical synchrony are particularly important for perception^35^.

### Astrocyte Adra1a receptor signaling shapes neuronal activity

**Fig. 6:**
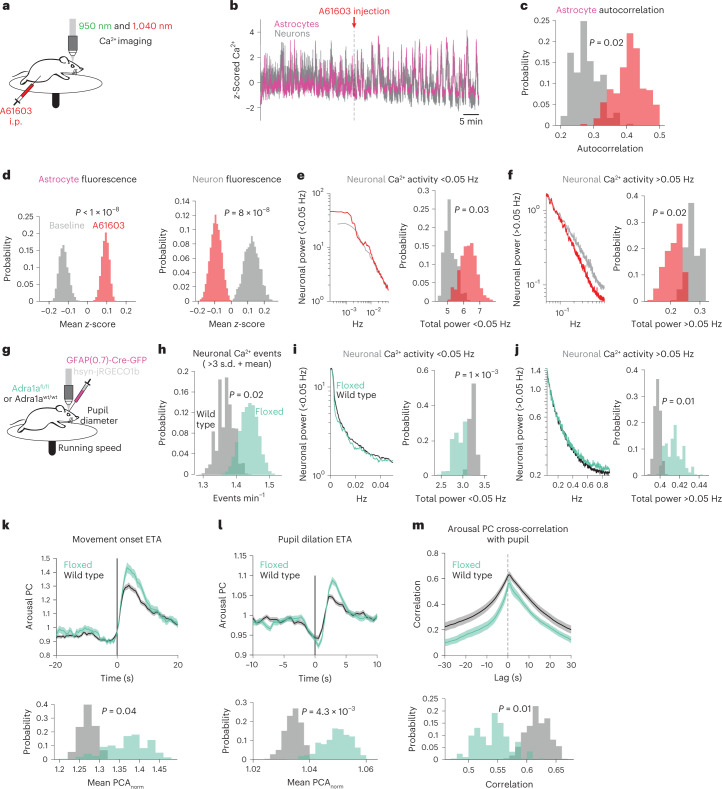
Adra1a receptors modulate basal neuronal activity and neuronal population responses to arousal. **a**, Experimental schematic for dual-color imaging with in vivo pharmacology. **b**, A striking example of population astrocyte (magenta) and neuron (gray) Ca^2+^ activity in response to A61603 (10 µg kg^−1^, i.p.). **c**–**f**, Histograms show differences between metrics at baseline (gray) or after A61603 (red) using HB (*n* = 4 mice in **c**–**g**): astrocyte Ca^2+^ activity became more homogeneous as measured by autocorrelation (**c**); astrocytes (left) showed increased population Ca^2+^ fluorescence while neurons (right) showed decreased population Ca^2+^ fluorescence after A61603 administration (**d**); neuronal activity power spectra <0.05 Hz (left) showed more power after A61603 injection (right, HB) (**e**); same as in **e** for power >0.05 Hz (**f**). **g**, Schematic for neuronal Ca^2+^ imaging in homozygous *Adra1a*^*fl/fl*^ mice and wild-type littermate controls; all mice were injected with GFAP-Cre (*n* = 4 *Adra1a*^*fl/fl*^ and four wild-type littermate controls in **h**–**l**). **h**, Overall, neuronal activity was increased in *Adra1a*^*fl/fl*^ mice (green) relative to wild type (gray). **i**,**j**, Neuronal activity <0.05 Hz was decreased and neuronal activity >0.05 Hz was increased in *Adra1a*^*fl/fl*^ mice. **k**, Arousal-associated neuronal activity was increased following movement in *Adra1a*^*fl/fl*^ mice (green, *n* = 177 movement bouts) compared with wild type (gray, *n* = 400). **l**, *Adra1a*^*fl/fl*^ mice (green, *n* = 923 pupil dilations) showed increased arousal-associated neuronal activity compared with wild type (gray, *n* = 1,396 pupil dilations) following stationary increases in pupil diameter. **m**, Arousal-associated neuronal activity was less correlated with the pupil diameter overall in *Adra1a*^*fl/fl*^ (green, *n* = 23 recordings) compared with wild-type mice (gray, *n* = 14 recordings). For all panels, histograms show HB distributions (*n* = 4 *Adra1a*^*fl/fl*^ and 4 wild-type littermate controls). All line plots are presented as mean ± s.e.m. and *P* values from histograms show one-sided HB tests.

On the basis of pharmacological and transcriptomic data, as well as its activation of astrocytes in other contexts^15–17,36^, we identified Adra1a receptors as a likely connector between astrocytes and cortical state. To test how Adra1a receptors modulated cortical activity, we injected the Adra1a receptor agonist A61603 (10 μg kg−1, i.p.)—which stimulates astrocytes in acute cortical slices^32^—while recording cortical astrocyte and neuron Ca^2+^ activity (Fig. 6a). We saw robust responses within minutes of A61603 administration (Fig. 6b). Astrocyte Ca^2+^ was more homogeneous (Fig. 6c) and elevated (Fig. 6d, left), while neuronal Ca^2+^ showed a general reduction in baseline Ca^2+^ (Fig. 6d, right), while exhibiting similar oscillatory activity as astrocytes. To better quantify these neuronal changes, we took the power spectrum of each neuron and separated the activity into slower (<0.05 Hz, Fig. 6e, left) and faster fluctuations (>0.05 Hz, Fig. 6f, left). We found that after A61603 administration there was more power in <0.05 Hz fluctuations (Fig. 6e, right) and less power in >0.05 Hz fluctuations (Fig. 6f, right). Our results indicate that A61603 administration, while increasing the coordination and level of astrocyte Ca^2+^ activity, broadly inhibits and alters the pattern of neuronal activity.

**Extended Data Fig. 6:**
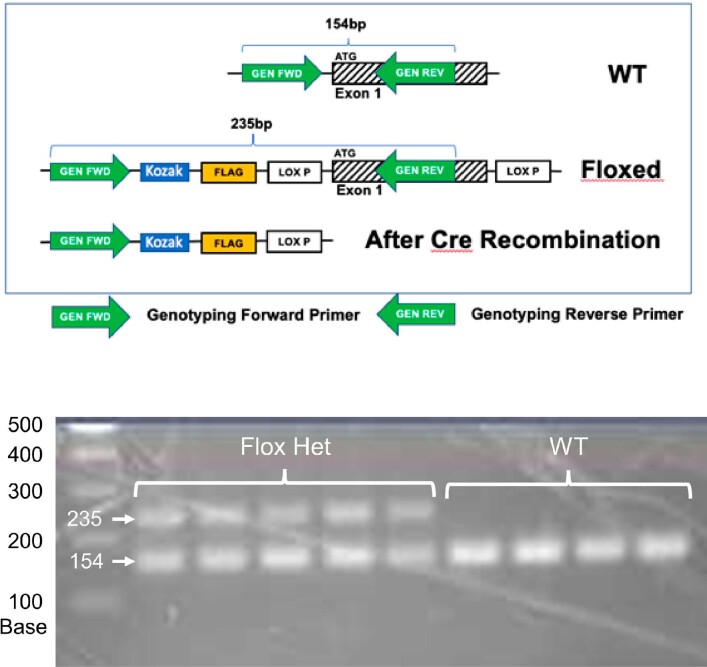
Generation of Adra1a^fl/fl^ mice. Top: schematic of the genetic strategy used to generate conditional deletion of Adra1a. Primers (green arrows) were designed to identify and distinguish between the endogenous and knock-in Adra1a alleles. Bottom: Numbers are in base pairs. Validation of the knock-in strategy used to generate Adra1a^fl/fl^ mice. PCR of Adra1a^fl/+^ mice generates both a 154 base pair band corresponding to the wild type allele and a 235 base pair band corresponding to successful LoxP knock-in, which is not found in wild-type mice. N = 5 Adra1a^fl/+^ and 4 wild type mice.

**Extended Data Fig. 7:**
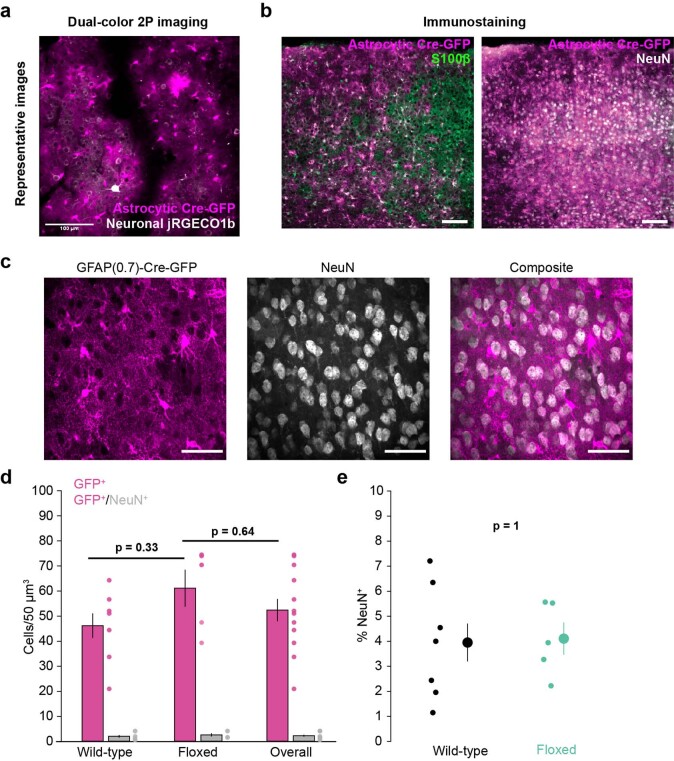
Astrocyte-specific Cre expression. **(a)** Representative average 2 P image of *in vivo* neuronal jRGECO1b (gray) and astrocytes expressing Cre-GFP (magenta) **(b)** Example images show astrocyte-specific Cre-GFP (magenta) expression overlaps with the astrocyte marker S100β (left, green) but not the neuronal marker NeuN (right, grey), scale bar = 100 µm. (c) Representative mean-projection of a confocal 60x z-stack used for cell counting. Each animal had two sections with each section having six distinct field-of-views containing 20 z-planes. GFAP(0.7)-Cre-GFP expression (left) was highly astrocytic and the neuronal marker NeuN (middle) was rarely colocalized (right, scale bars = 50 µm). **(d)** Cre expression in neurons (grey bars) was low across genotypes (overall = 2.2 ± 0.39 neurons/50 µm^3^) and no difference was found between Cre expression in Adra1a^fl/fl^ mice and control mice or the cohort as a whole (One-sided Kruskal-Wallis test). **(e)** Cre-expressing neurons were rare in both wild-type (black, 4.0% ± 0.75%) and Adra1a^fl/fl^ mice (green, 4.1% ± 0.65%, two-sided ranked-sum test). (n = 7 Adra1a^fl/fl^ and n = 5 wild-type mice for all graphs. Data are presented as mean ± S.E.M.).

**Extended Data Fig. 8:**
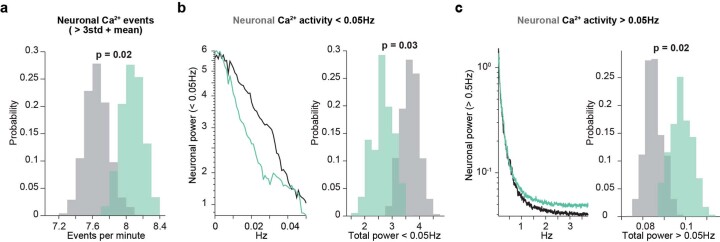
Resonant galvo imaging (7.5 Hz effective frame rate) confirms increased neuronal activity in Adra1a^fl/fl^ mice. **(a–c)** Histograms show neuronal activity in Adra1a^fl/fl^ (green) and wild-type littermate mice (grey) based on HB. **(a)** Neuronal Ca^2+^ event rate was increased in Adra1a^fl/fl^ (green, n = 4 mice) compared to wild-type mice (grey, n = 4 mice). **(b)** Left: power spectrum of Adra1a^fl/fl^ (green) and wild-type (black) Ca^2+^ activity between 0–0.05 Hz. Right: Total power in 0–0.05 Hz neuronal Ca^2+^ activity, comparing Adra1a^fl/fl^ and wild-type mice. **(c)** Same analysis as (b), comparing power in neuronal Ca^2+^ fluctuations > 0.05 Hz. All p-values from one-sided HB tests.

To determine how astrocytic Adra1a receptors specifically affect cortical neuronal activity, we generated a mouse line with LoxP sites flanking *Adra1a*, allowing for Cre-specific deletion of the receptor (Extended Data Fig. 6). We injected an astrocyte-specific Cre-GFP virus into the cortex of Adra1a^fl/fl^ mice (or wild-type littermate controls), and performed neuronal Ca^2+^ imaging while recording movement and pupillometry (Fig. 6g). In agreement with previous work^37^, we saw astrocyte-specific Cre expression which colocalized with the astrocytic marker S100β, but not the neuronal marker NeuN (Extended Data Fig. 7). Overall activity in these mice showed more neuronal Ca^2+^ events (Fig. 6h), as well as less power in <0.05 Hz Ca^2+^ fluctuations (Fig. 6i) and more power in >0.05 Hz Ca^2+^ fluctuations (Fig. 6j), the inverse of stimulating Adra1a receptors using A61603 (Fig. 6d–f). These results suggest NE signaling through astrocyte Adra1a receptors is a major pathway for modulating the level and pattern of neuronal activity. To ensure these results were not an artifact of imaging rate, we recorded neuronal activity using resonant galvanometers and saw the same results (Extended Data Fig. 8).

**Extended Data Fig. 9:**
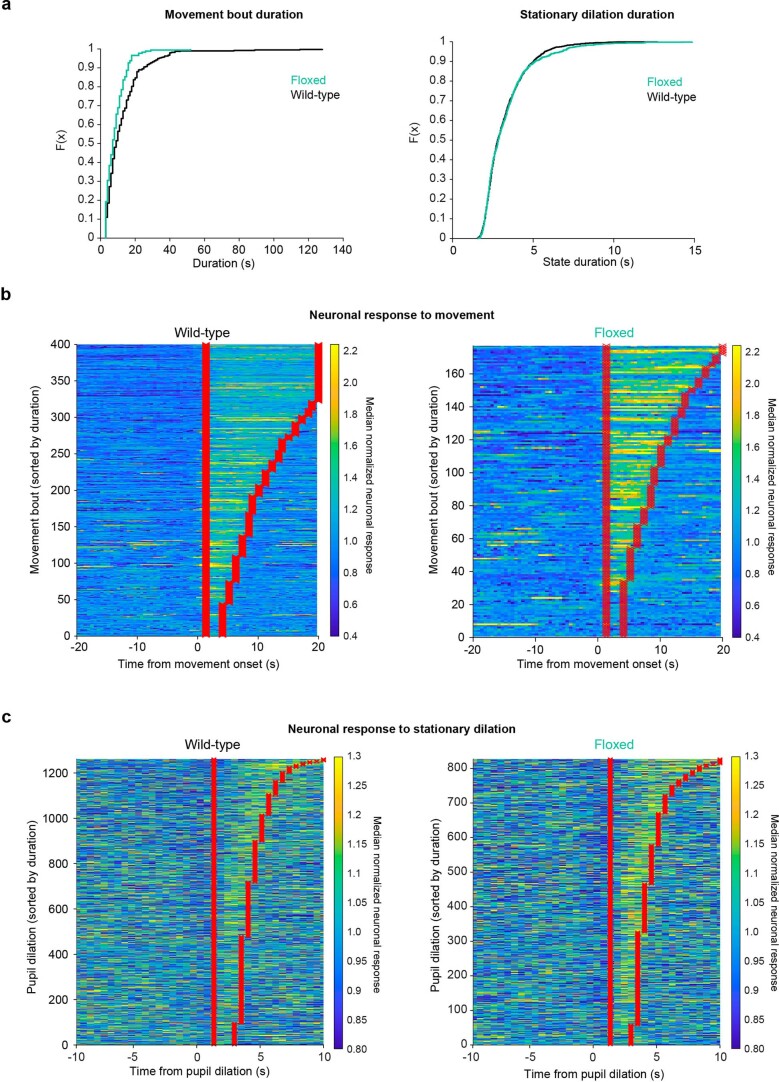
Quantification of arousal-associated neuronal activity. **(a**) Cumulative distribution plots of the duration of movement bouts (left) and stationary pupil dilations (right) for wild-type (black, n = 400 movement bouts and 1396 pupil dilations) and Adra1a^fl/fl^ (green, n = 177 movement bouts and n = 923 pupil dilations) mice. **(b)** Analysis range (red) used to quantify the normalized arousal PC response to movement in wild-type (left) and Adra1a^fl/fl^ (right) mice. Each row represents one movement event, and the movements from all recordings and mice are concatenated to show the entire dataset (n = 4 wild-type mice and n = 4 Adra1a^fl/fl^ mice). **(c)** Same analysis as in (b), showing stationary pupil dilations in Adra1a^fl/fl^ mice compared to wild-type.

We next determined whether astrocyte Adra1a receptors impacted arousal-related neuronal activity. As before (Fig. 3i), we assessed how arousal-related neuronal activity changed either with movement (Fig. 6k) or stationary pupil dilation (Fig. 6l). In this dataset, the distribution of durations during movement bouts (Extended Data Fig. 9a) and stationary pupil dilations (Extended Data Fig. 9b) was wide, and differed between *Adra1a*^*fl/fl*^ mice and wild-type controls. To account for this, we analyzed only arousal-related neuronal activity during the movement (Extended Data Fig. 9b) or pupil dilation event (Extended Data Fig. 9c), including a ~1 s offset to account for a delayed response in neuronal activity. In both cases, *Adra1a*^*fl/fl*^ mice displayed enhanced arousal-related neuronal activity (Fig. 6k,l). This increase could not be explained by an overall stronger neuronal connection to arousal; *Adra1a*^*fl/fl*^ neuronal activity was less correlated with pupil diameter than that in wild-type littermates (Fig. 6m). These results suggest that NE signaling through astrocyte Adra1a receptors regulates both the magnitude and shape of neuronal responses to arousal.

### NE signaling via astrocyte Adra1a modulates cortical state

**Fig. 7:**
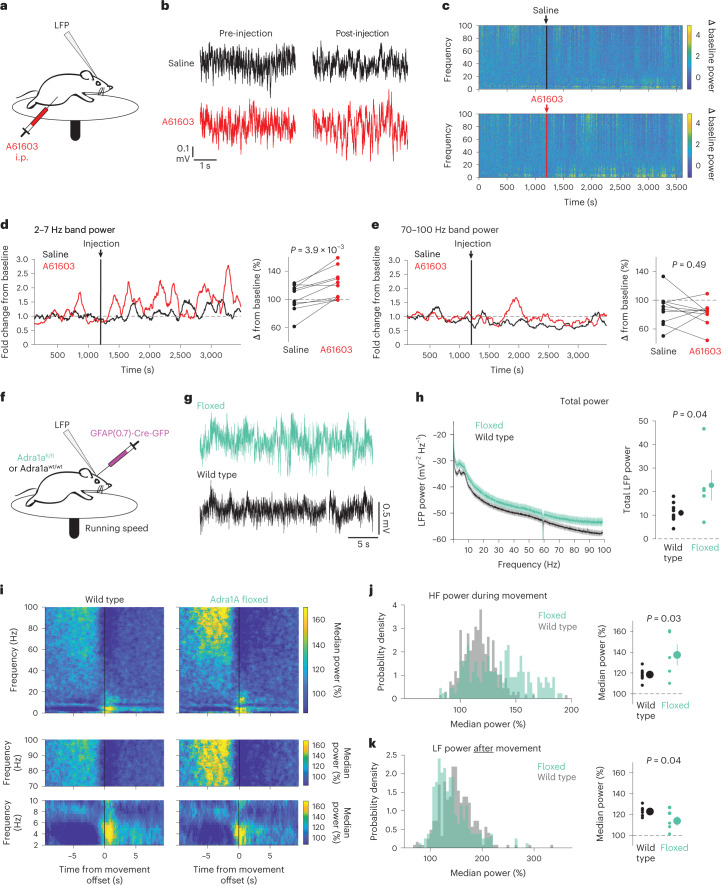
Genetic removal of astrocyte Arda1a impairs cortical resynchronization after arousal. **a**, Experimental setup for LFP recording with in vivo pharmacology. **b**, Example cortical LFP data following i.p. injections of either saline (black) or the Adra1a-specific agonist A61603 (1 µg kg^−1^, red). **c**, Representative spectrograms from saline-injected (top) and A61603 -injected (bottom) mice. Arrows and vertical lines indicate time of injection. **d**, Left: example of 2–7 Hz band power change with saline or A61603 injection, smoothed with a 2 min moving average. Right: A61603 increased LF power compared with saline (two-sided signed-rank test, *n* = 10 mice). **e**, Left: representative trace of 70–100 Hz band power with saline or A61603 injection. Right: A61603 did not affect HF power compared with saline (two-sided signed-rank test, *n* = 10 mice). **f**, LFP recordings were performed in homozygous *Adra1a*^*fl/fl*^ mice and wild-type littermate controls; all mice were injected with GFAP-Cre. **g**, Representative 30 s of LFP data from *Adra1a*^*fl/fl*^ (green) and wild-type (black) mice. **h**, Average LFP spectra from *Adra1a*^*fl/fl*^ (*n* = 5) and wild-type (*n* = 9) mice, with total LFP power higher in *Adra1a*^*fl/fl*^ mice (two-sided *t*-test, shaded region shows theoretical error bars with *P* = 0.05). **i**, Average LFP spectrograms around movement offset (*t* = 0 s) for wild-type (left) and *Adra1a*^*fl/fl*^ (right) mice. Data are normalized by the median power at each frequency to show state-related changes in LFP. Top: entire spectrograms from 0–100 Hz. Middle: 70–100 Hz range showing increased power in *Adra1a*^*fl/fl*^ mice compared with wild type during movement. Bottom: 2–7 Hz range showing reduced power after movement offset in Adra1a^fl/fl^ mice. **j**, There was increased 70–100 Hz power in *Adra1a*^*fl/fl*^ mice during movement (left, *n* = 303 wild-type and 194 *Adra1a*^*fl/fl*^ movement offsets) between cohorts (right, *n* = 5 *Adra1a*^*fl/fl*^ and 9 wild-type mice, two-sided *t*-test). **k**, Same as in **j** for 2–7 Hz power after mice stopped moving. For all scatter plots, individual data are plotted as mean ± s.e.m.

On the basis of the *Adra1a*-dependent relationship between astrocytes and cortical state, we hypothesized that, in contrast to the desynchronizing effect of NE on the cortex generally^11^, signaling through astrocytic Adra1a receptors would lead to cortical synchrony. We again injected the Adra1a receptor agonist A61603 (1 μg kg^−1^, i.p.) while recording cortical LFP (Fig. 7a). Compared with saline control, A61603 treatment resulted in high-amplitude LFP fluctuations (Fig. 7b) reflected by increased LF power (Fig. 7c,d). In contrast, we saw no significant difference in HF power (70–100 Hz, Fig. 7e). These effects show that, contrary to the role of NE more broadly^10,11,25^, stimulation of Adra1a receptors increases cortical synchrony.

However, the manipulation using A61603 was body-wide due to i.p. injection and affected all Adra1a-expressing cells. On the basis of our Ca^2+^ imaging results (Fig. 6g–m) in *Adra1a*^*fl/fl*^ mice, we wondered whether astrocytic Adra1a receptor signaling might modulate cortical state. To test this, we used the same receptor knockout strategy and recorded cortical LFP (Fig. 7f). In *Adra1a*^*fl/fl*^ mice, the cortical LFP was generally higher amplitude than in controls (Fig. 7g), with higher power across all frequency bands (Fig. 7h, left), and higher total LFP power (Fig. 7h, right). These results further support the idea that NE signaling to astrocytes modulates overall cortical neuronal activity.

We also hypothesized that removing astrocytic Adra1a receptors would effect NE-related cortical state transitions. We focused on times when mice stopped moving (Fig. 7i, top row), and found *Adra1a*^*fl/fl*^ mice had increased HF power during movement (Fig. 7i, middle row; Fig. 7j) and less LF power in the *Adra1a*^*fl/fl*^ mice compared with wild-type mice (Fig. 7i, bottom row; Fig. 7k). Taken together, these data show that selectively removing a NE receptor from astrocytes alters arousal-related cortical state changes, enhancing the desynchronizing effects of arousal and reducing resynchronization afterwards.

## Discussion

**Fig. 8:**
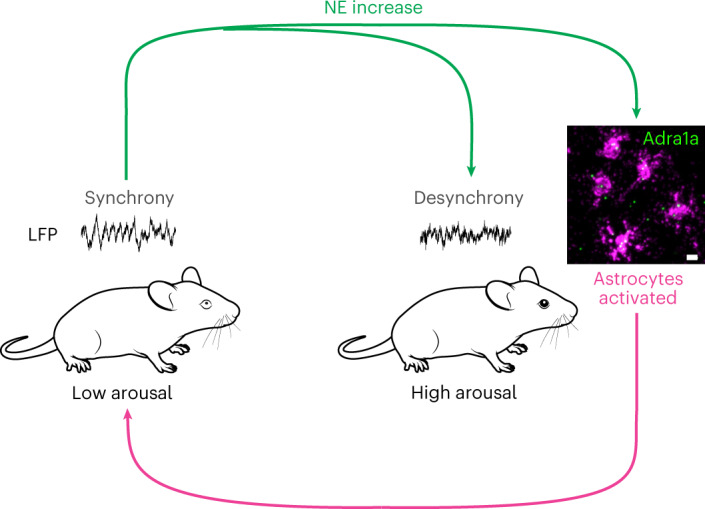
Model of astrocyte regulation of arousal-associated cortical state. NE (green arrows) drives changes from states of low arousal with synchronized cortical activity (left) to states of high arousal with desynchronized cortical activity (right). Simultaneous activation of astrocytes through the Adra1a receptor leads to Ca^2+^ signaling (magenta arrow) that drives the cortex back to a synchronized state following increases in arousal. Scale bar, 10 µm. Mouse images from SciDraw.io under a Creative Commons licence CC BY 4.0.

Understanding awake cortical state regulation is crucial for understanding perception, attention and behavior^1^. Most research examining cortical states has focused on arousal mechanisms—such as release of NE—that desynchronize the cortex and increase sensitivity to external stimuli^6,7,24^. However, we found that NE (Fig. 8, green) not only desynchronizes cortical neuronal activity, but also leads to cortical synchrony by activating astrocytic Adra1a receptors. This model identifies astrocytes as key regulators in the awake cortex, acting as a feedback mechanism for arousal-associated desynchrony (Fig. 8).

### Astrocytes regulate arousal-associated cortical circuits

We found that astrocytes responded more sensitively to arousal (Fig. 1) and NE (Fig. 2) than previously recognized^14–17^. Arousal-associated astrocyte Ca^2+^ activity occurred at the crux of reductions in arousal-associated neuronal activity (Fig. 3) and bi-hemispheric cortical state changes (Fig. 5), and was not dependent on local neuronal activity (Fig. 4). These results suggest direct NE signaling to astrocytes acts as a separate neuromodulatory pathway. To support this, we found that NE signaling through astrocyte Adra1a receptors regulates overall neuronal activity (Fig. 6h–j), the response of neurons specifically to arousal (Fig. 6k–m), and arousal-associated cortical states (Fig. 7f–k). Notably, we find that astrocytes detect relative changes in arousal, suggesting they act as a feedback mechanism without working against the arousal system more generally. This role may be particularly useful in the context of heterogeneous cortical NE dynamics. We observed extracellular fluctuations in NE^26^ on a timescale of seconds, similar to the reported activity patterns of the underlying locus coeruleus axons^28^, as well as much slower signaling on the order of tens of seconds to minutes (Fig. 2c). Thus, change detection may allow astrocytes to ignore the tonic fluctuations of cortical NE and sensitively respond to phasic increases in NE.

### Astrocyte Ca^2+^ is poised to regulate neuronal activity

Of course, astrocytes are not the sole detectors of cortical NE. Neurons, among other cell types^12,13^, also show strong responses to arousal^2,3,7,24^ and NE^10^. Understanding the relative effects of arousal on neuronal and astrocytic activity is crucial to understanding how these cellular populations contribute to arousal-mediated cortical state changes. Our results are notable in the context of previous research placing astrocyte activity downstream of neuronal activity, or to driving changes in neuronal activity on long timescales^32,38,39^. In contrast, our data show that arousal-associated astrocyte Ca^2+^ signaling occurs at the crux of the neuronal response to arousal, co-occurring with transitions in arousal-associated neuronal activity (Fig. 3). This relationship was also seen more broadly, with astrocyte Ca^2+^ occurring when bi-hemispheric arousal-associated desynchronized electrical activity decreased and cortical synchrony increased (Fig. 5). Furthermore, we found that NE-specific astrocyte Ca^2+^ signaling, and its relationship to arousal, was not dependent on the activity of nearby neurons, suggesting that astrocytes act directly downstream of neuromodulatory signaling to modulate neuronal activity (Fig. 4). These findings support a model in which astrocytes dynamically respond to NE to modulate neuronal activity on the physiological timescales of arousal.

### Receptor-specific relationship between NE and cortical state

Our work positions cortical astrocytes as drivers of neuronal synchrony on multiple scales in the wake state, and suggests new roles for NE in cortical state regulation. In contrast to the generally desynchronizing role of NE^11^, we found that specific pharmacological stimulation of the Adra1a receptor reduced cortical neuronal activity (Fig. 6d, right) and altered its pattern (Fig. 6e, f). Adra1a receptor stimulation also led to cortical synchrony increases without altering HF power (Fig. 7a–d). This relationship was initially hypothesized by early pharmacological work^40^, but to our knowledge has not been validated nor widely accepted. Our findings suggest that even within the same family, activation of different NE-receptor subtypes may have profoundly different effects on cortical state and by extension arousal, attention and behavior, which may help explain disparate effects of adrenergic signaling on perception and cognition^41,42^.

### What is arousal?

Here we used pupil diameter as an external read-out for a complicated set of biological processes that influence neural activity brain-wide^23,25,43,44^. While we focused on NE, many neuromodulators including acetylcholine^28,45^ and serotonin^46^ vary with arousal and modulate cortical state. Fully describing how neuromodulatory inputs affect astrocyte and neuronal activity will be vital for a complete description of cortical state regulation. Other internal states, such as motivation, interact with arousal and may involve neuromodulatory systems, but are not fully captured with the present arousal metrics. Here we were interested in pupil diameter and movement as external readouts of NE-related states, but future work should investigate the contributions of other internal states, using tools such as facial motion energy^29^, to further dissect astrocyte function in the context of internal states.

While our work establishes a role for astrocytes in mediating arousal-associated changes in neuronal activity and cortical state, we did not directly link these changes to behavioral outcomes. Future studies should focus on how arousal-associated astrocyte Ca^2+^ influences the general perceptual effects of arousal^7^ and extend these findings to the specific effects of attention^1,35,43,47^. It will also be important to examine how sensory stimuli-driven^48–50^ and arousal-driven astrocyte Ca^2+^ signaling leads to changes in behaviorally relevant neuronal population activity and cortical synchrony.

### How does arousal modulate different cellular subpopulations?

To expand the impact of our findings, the experiments described here could be extended to dissect the role of specific subpopulations of neurons and astrocytes, or types of Ca^2+^ activity in these populations. In this work, we analyzed Ca^2+^ imaging data as homogeneous cellular populations, in part due to an acquisition rate (~2 Hz) necessary for imaging astrocyte populations on a wider scale (0.5 mm^2^). However, there may be differences between the population-level Ca^2+^ activity we identified and fast Ca^2+^ responses to arousal.

Both astrocytes^34,51^ and neurons^52^ can be split into multiple subpopulations, and neuronal subtypes—in particular inhibitory interneuron subtypes—have unique relationships to arousal and cortical state^53^. In our work, we used PC analysis to account for heterogeneous neuronal responses to arousal, and we found Adra1a-mediated astrocytic modulation of neuronal responses to arousal. However, we did not identify any specific effects on neuronal subtypes. On the basis of the overall reduction of neuronal activity with A61603 injection (Fig. 6d, right), and the overall increase in neuronal activity (Fig. 6h) and LFP power with knockout of astrocytic-Adra1a receptors (Fig. 7h), our work suggests astrocytic modulation of cortical inhibition is a likely candidate for the downstream circuit mechanisms controlling cortical state, as has been proposed in other contexts^36,54^. Cortical interneurons have been shown both to play a critical role in the control of cortical state^45,55,56^, and to be particularly sensitive to their electrostatic extracellular environment^57^. Neuromodulator-driven astrocytic modulation of the extracellular environment^58^ and inhibitory regulation of nearby neurons^18^ may act as prime loci of control for cortical circuit activity and state.

Within our smFISH dataset (Extended Data Fig. 5) and in published single-cell data^51^, cortical astrocytes show variable expression of neuromodulatory receptors, and some astrocytes lack any transcripts for Adra1a. This suggests molecularly distinct, and potentially functionally distinct, subpopulations of cortical astrocytes with differential sensitivity to arousal-related neuromodulators. It also suggests that gap junctions, ATP signaling and other mechanisms^59,60^ that create networks within the astrocyte syncytium may be important for arousal-associated astrocyte activity and modulation of cortical circuits. Identifying the specific relationships among subpopulations of astrocytes and neurons, and determining the manner and magnitude of their modulation by arousal, will be important for understanding what constitutes a cortical circuit and how cortical circuit activity is regulated. Furthermore, much like the effects of arousal, these relationships may be layer^35^ or cortical area^44^ specific. Future study of cellular subpopulation-specific effects on cortical state, particularly in additional cortical regions and in combination with behavioral assessments, will provide a richer appreciation of the cellular mechanisms that regulate arousal-associated cortical state.

## Methods

### Animals

All procedures were carried out in accordance with protocols approved by the University of California, San Francisco Institutional Animal Care and Use Committee. Animals were housed and maintained in a temperature-controlled environment on a 12 h light–dark cycle, with ad libitum food and water. Male and female mice were used whenever available. All imaging/electrophysiology experiments were performed at the same time each day. Adult C57BL/6 mice, *Adra1a*^*fl/fl*^ mice or *Adra1a* wild-type mice aged 1–6 months at time of surgery were used. For experiments involving *Adra1a* ^*fl/fl*^ mice, the experimenter was blind to animal genotype before surgery, recording and analysis.

#### Generation of Adra1a^fl/fl^ mice

The Mouse Biology Program at the University of California, Davis, constructed the mouse. A floxed FLAG-α1A knock-in vector was made using standard methods as follows: The targeting vector contained a 5′ arm of 5.4 kb and a 3′ arm of 5.5 kb. Lox P sites were placed upstream and downstream of the α1A-AR gene first coding exon. A Kozak sequence and single FLAG tag were upstream, and the neomycin-resistance gene was downstream.

The vector was electroporated into the C57BL/6N ES cell line JM8.F6. The resulting ES cell clones were screened by long-range PCR for loss of the native allele and homologous recombination, and containing a single copy of the plasmid by LoxP PCR. Two clones passed these screens and had normal chromosome counts. Both ES clones were microinjected into blastocysts, and transferred to embryonic day 2.5 stage pseudo-pregnant recipients. The resulting chimeras were screened for percent ES cell derived coat color, and those greater than 50% were mated to C57BL/6N females. Germline heterozygous mice were produced, and mated to MMRRC strain C57BL/6-Tg(CAG-Flpo)1Afst/Mmucd, RRID: MMRRC:036512-UCD, for excision of the Neo selection cassette. Neo-excised mice were identified via PCR, and mated further to C57BL/6J mice to remove the FLP transgene. Mice were continued in C57BL/6J.

Routine PCR genotyping used 5′-gcttcctcaggctcacgtttcc and 3′-gccttagaaatgttcacctgtgc primers upstream and downstream of the LoxP site (Extended Data Fig. 6). These mice are available upon request from the corresponding author.

### Surgical procedures and viral infection

Mice were administered dexamethasone (5 mg kg^−1^, subcutaneous) at least 1 h before surgery, and anesthetized using 1.5% isoflurane (Patterson Veterinary Supply, 78908115). After hair removal and three alternating swabs of 70% ethanol (Thermo Fisher Scientific, 04-355-720) and Betadine (Thermo Fisher Scientific, NC9850318), a custom-made titanium headplate was attached to the skull using cyanoacrylate glue and C&B Metabond (Parkell, S380). If recording LFP, 0.5 mm burr holes were made bilaterally over the visual cortex, and bilaterally over the cerebellum for reference, and a ~200-µm-diameter perfluoroalkoxy-coated tungsten wire (A-M Systems, 796500) was implanted -0.2 mm into each hole and secured with Metabond. For imaging and LFP experiments, a 3 mm craniotomy was made over the right visual cortex and the right visual cortex burr hole was made lateral to the craniotomy.

For viral infection, 400–800 nl total volume of the following viruses were injected alone, or in combination by premixing the solutions before aspiration: AAV5.GfaABC_1_D.GCaMP6f.SV40 (Addgene, 52925-AAV5), AAV9.hSyn.NE2h (WZ Biosciences, YL003011-AV9), AAV9.GfaABC_1_D.jRGECO1b (Vigene, custom-ordered), AAV9.Syn.GCaMP6f.WPRE.SV40 (Penn Vector Core, AV-8-PV2822), AAV2.hSYN.hM4D(Gi).mCherry (Addgene, 50475-AAV2) and AAV5.GFAP(0.7).EGFP.T2A.iCre (Vector Biolabs, VB1131).

Injections were made through a glass pipette and UMP3 microsyringe pump (World Precision Instruments) into one or two locations in the right visual cortex at coordinates centered on +2.5 mm medial/lateral, +0.5 mm anterior/posterior and −0.3 dorsal/ventral from lambda. After allowing at least 10 min for viral diffusion, the pipette was slowly withdrawn and a glass cranial window implanted using a standard protocol^61^.

For in vivo fiber photometry recordings, GRAB_NE_2h (AAV9.hSyn.NE2h) and astrocytic jRGECO1b (AAV9.GfaABC_1_D.jRGECO1b) were expressed via viral vectors in C57Bl/6 mice. A 1-mm-diameter craniotomy was made over the PFC (+1.7–1.8 mm rostral, +0.5 mm lateral from bregma), and viral vectors were delivered to −2.3 to 2.4 mm ventral. A fiber optic cannula (Mono Fiberoptic Cannula, 400 µm core, 0.66 NA, 2.8 mm length, Doric Lenses) was then lowered to −2.3 mm ventral and secured in place using dental cement.

### Immunohistochemistry and in situ hybridization

#### Immunohistochemistry

Following in vivo experiments, mice were overdosed on isoflurane and then perfused transcardially with phosphate-buffered saline (PBS, Sigma-Aldrich P3813) followed by 4% paraformaldehyde in PBS (Santa Cruz Biotechnology, CAS 30525-89-4). Brains were removed and postfixed overnight in 4% paraformaldehyde, followed by cryoprotection in 30% sucrose in PBS for 2 days at 4 °C. Brains were snap frozen in dry ice and stored at −80 °C until adhesion onto a sectioning block with Optimal Cutting Temperature Compound (Thermo Fisher Scientific, 23-730-571). Forty-micrometer coronal sections were taken on a cryostat and stored in cryoprotectant at −20 °C until immunohistochemistry was performed. For immunohistochemistry, free-floating sections spanning the rostral–caudal axis were selected and washed with 1× PBS for 5 min three times on an orbital shaker, followed by permeabilization with 0.01% PBS-Triton X for 30 min. Sections were then blocked with 10% NGS (Sigma-Aldrich, S26-100ML) for 1 h. Immediately after, sections were incubated with chicken α-GFP (1:3,000, Abcam, ab13970) and either rabbit α-NeuN (1:1,000, EMD Millipore, ABN78) or mouse α-NeuN (1:1,000, Millipore Sigma, MAB377) and rabbit α-S100B (1:500, Millipore Sigma, SAB5500172). Sections were then washed with 1x PBS for five minutes three times on a shaker, followed by secondary incubation with Thermo Fisher Scientific goat α-chicken Alexa Fluor 488 and either goat α-rabbit Alexa Fluor 405 or goat α-mouse Alexa Fluor 405; goat α-rabbit Alexa Fluor 555 for 2 h at 20 °C on a shaker. Sections were washed again with 1× PBS for 5 min three times, then mounted and cover slipped with Fluoromount-G. For whole section examples, images were taken using a Keyence BZ-X800 fluorescence microscope to assess viral spread. Then, 2× images were acquired and the images were computationally stitched with Keyence Analysis Software. For cell counting 60× *z*-stacks were captured on a spinning-disk confocal (Zeiss). Slides were oil-immersed. The Fiji plugin Cell Counter was used to quantify the number of GFP^+^, NeuN^+^ and GFP^+^/NeuN^+^ cells to determine colocalization. Each animal had two sections, with each section having six distinct field of views containing 20 *z*-planes. A cell was considered GFP^+^ when signal was confined to soma and processes, and GFP^+^/NeuN^+^ when cells exhibited merged signals.

#### Single-molecule fluorescent in situ hybridization

Single-molecule, fluorescent in situ hybridization data collection was performed using LaST map smFISH as previously described^34^. Astrocyte cell boundaries were segmented using an ilastik pixel classifier and a customized watershed segmentation. To preserve processes of astrocyte and not cut them off from the DAPI signals, a pixel classifier was trained by using only large Gaussian filters (5/10 pixels) during the feature extraction step. As a result, astrocyte processes were detected with fewer splits. Pixel classification carves out only non-background pixels from the image and does not identify the astrocyte boundaries separating neighboring cells, that is, instance segmentation. To address this problem, we first used CellPose to identify all nuclei from the image. Subsequently, the centroids of all nuclei were extracted and used as the seeds to generate astrocyte boundaries between adjacent cells using a watershed segmentation. As a result, astrocytes with touching processes were separated. Finally, as non-cell debris might remain in the segmentation image, we further used an object classification workflow in ilastik to remove them.

All mRNA signals were detected using a Python package called TrackPy using five pixels as diameter and 96 as percentile threshold. These detected spots were then assigned to each astrocyte that they sat within by using a Python package called shapely. Due to the tissue damage that occurred during the sample preparation step, only seven cortex surface areas from all sections were eligible for downstream processing to ensure the comparability between tissue sections. For all these regions, both white matter boundaries and the superficial cortex area were manually annotated. For each cell, the distance to both the white matter boundary (d_WM) and distance to the cortex surface (d_ep) were calculated. The relative cortical depth (D_relaCortex) was thus defined as:

$$D_{{\mathrm{relaCortex}}} = \frac{{d_{{\mathrm{ep}}}}}{{d_{{\mathrm{ep}}} + d_{{\mathrm{WM}}}}}$$

#### Recording setup

Animals were given at least 1 week after surgery for recovery and viral expression. They were then habituated on a custom-made circular running wheel over at least 2 days, and for a cumulative time of at least 2.5 h, before experimental recordings began. After habituation, mice were head-fixed on the wheel and movements were recorded by monitoring deflections of colored tabs on the edge of the wheel using an optoswitch (Newark, HOA1877-003).

#### Pupillometry

Pupil recordings were made using a Genie near-infrared camera (1stVision, M640) and a telescopic lens (Thorlabs, MVL50TM23), and acquired at 30 Hz using the MATLAB Image Acquisition toolbox. A small monitor (Amazon, B06XKLNMW3) showing a consistent teal background color (RGB: 0,1,1) was placed by the mouse to allow for observation of the full range of pupil dynamics in an otherwise dark room. For experiments without 2P illumination, a near-infrared light (Amazon, B00NFNJ7FS) was used to visualize the pupil.

#### 2P imaging

2P imaging was performed on a microscope (Bruker) with two tunable Ti:sapphire lasers (MaiTai, SpectraPhysics) and a Nikon 16×, 0.8 numerical aperture water-dipping objective with a 2× optical zoom (frame rate 1.7 Hz, field of view 412 µm^2^, resolution 512 × 512 pixels). A 950 nm excitation light with a 515/530 emission filter was used to image green-emitting fluorophores, and 1,040 nm light with a 605/615 emission filter was used to image red-emitting fluorophores. Recordings lasted from 10 min to 1 h.

#### Electrophysiology

Visual cortex LFP was recorded at 1 kHz and subtracted from the ipsilateral cerebellar LFP before 1 kHz amplification (Warner Instruments, DP-304A) and acquired using PrairieView (Bruker) or PackIO^62^.

#### In vivo pharmacology

Recordings were taken before and after saline, Prazosin–HCl (5 mg kg^−1^ Sigma-Aldrich, P7791-50MG), A61603(1 µg kg^−1^ or 10 µg kg^−1^, Tocris, 1052), or clozapine N-oxide (CNO, 1 mg kg^−1^ or 5 mg kg^−1^, Tocris, 4936) were injected intraperitonially while animals remained head-fixed on the wheel, to ensure post-treatment recordings were comparable with baseline measures.

### Fiber photometry

Dual-color fiber photometry recordings were performed on a Tucker-Davis Technologies RZ10X processor with 405, 465 and 560 nm LEDs. LED drivers were modulated such that light power was approximately 15 μW for 405 nm and 20 µW for 465 nm and 560 nm wavelengths at the tip of the light path. Animals were recorded in a freely moving arena in which the mouse was able to move in all directions, after coupling to low-autofluorescence fiberoptic patchcords connected to photosensors through a rotary joint (Doric Lenses). Fluorescence signals were recorded for 10 min, during which tail lifts were performed every 2 min. For a tail lift stimulation, the experimenter held and lifted the tail of the animal until its hind paws disconnected from the ground; after that the tail was released. With this experimental paradigm, no pain or harm is caused to the animal.

### Statistics and reproducibility

All data analysis was done in MATLAB unless otherwise indicated. No statistical methods were used to predetermine sample sizes, but they are similar to previous reports^14,28^. Box plots are shown with the central mark indicating the median and the bottom and top edges of the box indicating the 25th and 75th percentiles, respectively. Whiskers extend to the most extreme data point or within 1.5 times the interquartile range (IQR) from the bottom or top of the box, and all other data are plotted as individual points, as listed in the figure legends. For statistical comparisons, nonparametric tests were used, or where indicated, normality was assumed but not formally tested, and *t*-tests were used. HB was performed on the basis of a MATLAB implementation (https://github.com/jenwallace/Hierarchical_bootstrap_Matlab) of the methodology, and used to reduce the statistical error rate of comparisons while retaining statistical power^63^. All multiway comparisons were adjusted for using Tukey–Kramer correction. No data were excluded from analyses except for the following (not predetermined): In hSYN-hM4Di experiments, outliers were excluded across all conditions from small stationary responses to avoid confounding effects from other influences on astrocyte Ca^2+^, as described in methods. For in vivo pharmacology experiments, electrical artifacts in band power were excluded before analysis, as described in methods. Samples were allocated into experimental groups by cell-type expression of each individual fluorescent sensor. Only adult animals (1–6 months of age) were used in experiments, and both males and females were used and randomly selected. For imaging and electrical recordings of spontaneous activity, blinding was not relevant because cell-type viral expression is evident from expression pattern. For in vivo pharmacology, blinding was not possible because control recordings were taken before treatment recordings to avoid confounding the treatment effects. For *Adra1a*^*fl/fl*^ mice, the experimenter was blinded to genotype before data collection and analysis.

#### Speed calculations

To compute wheel speed, a detected break in the optoswitch circuit was determined when the absolute value of the derivative of the raw voltage trace was at least 2 s.d. above the mean. For recordings with very little movement (s.d. <0.1), this threshold generated false positives so a set threshold of 0.1 was used. The number of breaks in the optoswitch circuit per second was then calculated, and using the circumference and number of evenly spaced colored tabs at the edge of the wheel, the wheel speed was determined and used for all subsequent analyses using speed. Movement periods were defined by wheel speed ≥10 cm s^−1^, and movement bouts that were separated by ≤2 s were considered one event. To ensure that movement-related dynamics were not included in stationary analysis, data were excluded from at least 10 s around identified movement periods.

#### Pupillometry

Following acquisition, pupil data were processed through a Python function that used contrast detection to identify the edges of the backlit pupil from the sclera and fit an ellipse whose major radius was taken as the pupil diameter. The diameter was then low-pass filtered to 0.5 Hz and normalized to a range between 0 and 1 to give pupil diameter as percent maximum. The pupil derivate was normalized to the acquisition rate (30 Hz) to compute pupil phase and to determine the phase of astrocyte Ca^2+^ events and the cross-correlations with GRAB_NE_. Stationary arousal dilations and constrictions were identified by the sign of the calculated pupil derivative, and only changes in pupil diameter >10% were used for subsequent analyses.

#### LFP

All spectral analysis was done using Chronux^64^. Raw LFP data were visually inspected to confirm useable signal was present, and then 60 Hz noise was filtered out and drifting baselines were compensated for using linear fitting. LFP power for frequency bands was computed using built-in Chronux functions with a time bandwidth of 2.5 and two tapers, no frequency padding and 5 s moving windows. For changes in LFP band power around arousal or astrocyte Ca^2+^ events, the median band-limited power was obtained and then normalized to the median band-limited power in the event-triggered time window to get relative band power. The median power before an event onset versus after was combined for each recording, and for *Adra1a*^*fl/fl*^ mice, the ratio between the two was computed and compared with *Adra1a* wild-type mice. For changes in LFP power after A61603 administration, the band-limited power for saline and drug data was calculated, outliers were removed to avoid contamination by recording artifacts, and then this power was normalized to the band-limited power for the respective baseline recording.

For total power in *Adra1a*^*fl/fl*^ and control mice, no baseline correction was done to avoid skewing the analysis. Spectrum power was calculated by concatenating recordings from all mice of each genotype and computing average or individual spectra with a time bandwidth of six and eight tapers to increase accuracy. The total power was then computed by summing across all frequency bands. Relative power was computing by dividing the spectrum from each mouse by its total power, and relative band power was computed by summing the power from each frequency band and dividing by the total power.

#### Ca^2+^ imaging

Astrocyte Ca^2+^ events and fluorescence was extracted using the AQuA software analysis package^22^. For dual-color imaging with neuronal Ca^2+^ indicators, particular care was taken to avoid AQuA detection of neuronal activity; the s.d. of the neuronal channel was taken in FIJI and a mask was created in AQuA to exclude areas of high neuronal activity and soma from analysis. Astrocyte events were included only if they had an area greater than 10 µm, lasted for at least two frames and had an AQuA *P* value <0.05. To obtain the average astrocyte Ca^2+^ fluorescence, the compensated fluorescence traces that account for spatially overlapping events were taken, normalized to their maximum value and then averaged together.

Neuronal Ca^2+^ events and fluorescence were extracted from neuropil background semi-automatically using Suite2P^65^. We identified Ca^2+^ events by taking identified spikes in the Ca^2+^ fluorescence data and thresholding them for only the largest (>3 s.d. over the mean) events. To calculate the average neuronal Ca^2+^ fluorescence, the trace from each neuron was normalized to its maximum value and averaged together.

#### Machine-learning based analysis of input contribution to astrocyte Ca^2+^

As an alternative to assess the contribution of biological inputs on astrocyte Ca^2+^, data from dual-color Ca^2+^ imaging were used to train a machine-learning model to predict average Ca^2+^ activity. To include LFP data, the spectrogram data from each LFP recording were decomposed using PC analysis based on the eigenvectors from the ipsilateral recording that accounted for the largest proportion of the variance. The PC1 in this data corresponded to cortical synchrony, with positive weights for HF and negative weights for low frequencies, matching a previous report but with inverse sign^66^. LFP PC1 for ipsilateral and contralateral recordings, as well as speed, pupil diameter and average Ca^2+^ fluorescence for neurons and astrocytes, was then *z*-scored and resampled to 10 Hz before being concatenated. This dataset was then imported into Python for machine learning analysis using the SciKit-learn toolbox. For analysis, randomly generated data were added for comparison, and rows without both ipsilateral and contralateral LFP recordings were excluded from subsequent analysis. Average astrocyte Ca^2+^ data were used as the target dataset and data were split into training (80%) and testing (20%) before classification using a random forest regression model. The model was validated using the *R*^2^ between the predicted average astrocyte Ca^2+^ fluorescence from the model and the actual average astrocyte Ca^2+^ data of the test set. Permutation testing of the predictors was then used to determine their relative contributions to model prediction.

#### GRAB_NE_ analysis

In GRAB_NE_ imaging data, we observed background fluorescence fluctuations that we thought might arise from hemodynamic artifacts. To ensure the data reflected the NE signal, hemodynamic artifacts in the data were removed by a custom-designed, data processing pipeline. The predominant hemodynamic artifact in the data was assumed to reflect fluctuating hemoglobin levels altering brain absorptivity causing an attenuation of light. As such, the signal from each pixel could be modeled as

$$Y_k\left( t \right) = F_k\left( t \right) \cdot e^{ - \mu _k\left( t \right)X} + N\left( t \right) = F_k\left( t \right) \cdot e^{h_k\left( t \right)} + N\left( t \right)$$

where *k* is index of pixel, *Y*_*k*_(*t*) and *F*_*k*_(*t*) are respectively the observed curve and the real fluorescence of *k*th pixel, *μ*_*k*_(*t*) is the absorption coefficient for *k*th pixel, *X* is the path distance, the term $e^{h_k\left( t \right)}$ represents the intensity attenuation, and *N*(*t*) is the noise. In our data, the identified hemodynamic signal across pixels was approximately synchronous but varied in magnitude; thus, the attenuation of one pixel can be represented by another, that is $e^{h_j\left( t \right)} = e^{ah_k\left( t \right) + b}\left( \,{j \ne k} \right)$. On the basis the findings above, we designed the following pipeline:

1. We selected one connected vascular region with minimal fluorescence in the average projection of the data and calculated an initial vascular reference curve. This region was assumed to have the lowest possibility to contain any true GRAB_NE_ signal.
2. To avoid compensating for slow changes in the true GRAB_NE_ signal, we subtracted the curve of each pixel by a 100-frame moving average.
3. We applied linear regression and fit the logarithm of the processed curve to the initial vascular reference curve. The exponential of the fit data was then taken to represent the hemodynamic effect for each pixel. To account for cases where the initial reference curve was contaminated, we iteratively refined the reference curve before fitting, that is, we calculated the weighted average of the original curve for all pixels (fitting parameter *a* in $e^{ah_k\left( t \right) + b}$ for each pixel is considered as the weight) and subtracted its moving average.
4. We removed the hemodynamic artifact (if any) by dividing the raw pixel curve by the exponential of the fitting data, $F_k\left( t \right) \approx \frac{{Y_k\left( t \right)}}{{e^{h_k(t)}}}$.

Next, only the least (1–25%) hemodynamically affected pixels with the lowest *a* were taken, excluding the bottom 1%, which often contained artifacts, and these were averaged together and used as the final GRAB_NE_ signal. For spectral analysis, each recording was concatenated together in 10 min segments, padding with its median value if necessary, and then run in Chronux with no frequency padding, a time bandwidth of 3, five tapers, and passing frequencies above half the window size (3 × 10^−3^ Hz) and less than the Nyquist frequency (0.9 Hz). To identify phasic increases in the GRAB_NE_ signal, built-in MATLAB functions were used to determine local peaks in the signal and a prominence threshold was used to determine the different magnitudes of GRAB_NE_ increases.

#### Event-triggered averages

All averages were computed by identifying events (for example, movement offset, pupil dilation and so on) and taking data in a symmetric time window around the events. The data are subsequently plotted as the mean and standard error across all events, except for spectrograms where the median was used.

#### Correlations

For comparisons of maximum and change in astrocyte Ca^2+^/pupil/speed after arousal, values were computed for each trace separately and then linearly correlated with *P* values describing the probability of a true *R*^2^ relationship between each two metrics. For correlations between astrocyte and neuronal Ca^2+^ activity, the average population fluorescence was taken for each and *z*-scored before cross-correlation. For behavioral state-separated cross-correlations, the same procedure applied, but only *z*-scored data from either moving periods or stationary periods were used. For correlations within neuronal and astrocyte populations, the pairwise correlation between each cell (neurons) or event (astrocytes) was computed, the symmetric and autocorrelations were excluded, and the overall mean was taken to obtain a single value indicating the synchrony of Ca^2+^ dynamics within each cellular population. For cross-correlations between pupil and imaging data (GRAB_NE_ and astrocyte Ca^2+^), all data were *z*-scored, resampled to either 10 or 30 Hz, padded with nan values if unequal in length, and then cross-correlated. For cross-correlations between LFP band power and Ca^2+^ imaging data, all data were *z*-scored, averaged and then resampled to 2 Hz to match the LFP resolution before cross-correlation.

#### hSYN-hM4Di

To estimate the effect of CNO on astrocyte Ca^2+^, the average Ca^2+^ event properties and overall event rate for each recording were randomly sampled 10^4^ times and CNO data were subtracted from corresponding baseline data. This procedure generated the range of treatment effects possible from the sampled data, and a *P* value was calculated as the proportion of CNO difference from baseline that was less than the maximum, or greater than the minimum, difference found in saline conditions. For calculating the modulation of astrocyte Ca^2+^ responses to arousal, the absolute change in average astrocyte Ca^2+^ fluorescence after either movement onset or pupil dilation was determined. Outliers during small stationary responses, which might reflect the influence of other variables on astrocyte Ca^2+^, were excluded. The magnitude of astrocyte Ca^2+^ responses to arousal after treatment was then compared with baseline responses.

#### Neuronal arousal PC analysis

PCA was done using the built-in MATLAB function on *z*-scored neuronal Ca^2+^ data. The pupil diameter and PC data were then resampled to an effective rate of 10 Hz, and the Pearson’s correlation between the pupil diameter and each PC was used to identify the arousal PC for each recording This PC was then normalized to the maximum value before subsequent analysis.

For comparisons between wild-type and *Adra1a*^*fl/fl*^ mice, the response to each movement onset or stationary pupil dilation was normalized to the median value in the window, and then the average arousal PC value during the event period was taken with a two-frame offset to account for a slight lag in the arousal-associated neuronal response.

#### Fiber photometry

Photometry data were detrended using linear regression to correct bleaching and normalized by *z*-scoring. For startle experiments, recordings were denoised using a FIR filter (cutoff at 2 Hz, transition width 0.5 Hz). Startle responses were aligned to the onset of the jRGECO signal by fitting a sigmoid to the evoked jRGECO and taking the fourth derivative to identify the onset inflection point.

### Reporting summary

Further information on research design is available in the Nature Portfolio Reporting Summary linked to this article.

## Online content

Any methods, additional references, Nature Portfolio reporting summaries, source data, extended data, supplementary information, acknowledgements, peer review information; details of author contributions and competing interests; and statements of data and code availability are available at 10.1038/s41593-023-01284-w.

## Supplementary information

Supplementary Information Supplementary Tables 1–5.

Reporting Summary

Supplementary Video 1 **2P imaging of GRAB**_**NE**_ **and astrocyte jRGECO1b**. Thirty-minute recording of spontaneous GRAB_NE_ (green) and astrocyte jRGECO1b (magenta) fluctuations in layer 2/3 of visual cortex of an awake mouse. Recording was acquired with a ~410 µm × 410 µm field of view, 10× averaged, and a one-pixel median filter was applied. Video is played at a 10 Hz frame rate.

Supplementary Video 2 **2P imaging of neuronal GCaMP6f and astrocyte jRGECO1b**. Ten-minute recording of spontaneous neuronal GCaMP6f (gray) and astrocyte jRGECO1b (magenta) fluctuations in layer 2/3 of the awake cortex. Recording was acquired with a ~410 µm × 410 µm field of view, 10× averaged, and a 0.5-pixel median filter was applied. Video is played at a 10 Hz frame rate.

Supplementary Video 3 **Neuronal and astrocyte Ca**^**2+**^ **responses to** **A61603**. Neuronal GCaMP6f (gray, left) and astrocyte jRGECO1b (magenta, right) recorded for 30 min at baseline and for 40 min post-injection of A61603 (10 µg kg^−1^, i.p.) Labels on the top left indicate pharmacological condition. Recording was acquired with a ~410 µm × 410 µm field of view, 10× averaged, and a 0.5-pixel median filter was applied. Video is played at a 60 Hz frame rate.

Supplementary Video 4 **2P** ***z*-stack showing neuronal hM4Di and astrocyte GCaMP6f expression in same tissue region**. An ~300 µm *z*-stack of in vivo expression of neuronal hM4Di-mCherry (gray) and astrocyte jRGECO1b (magenta). The *z*-stack was acquired with a 5 µm step size. A four-frame moving average projection (astrocytes) or s.d. projection (neurons) was calculated for a display step size of 20 µm per frame. Neuronal data were background-subtracted to aid visualization of soma, and astrocyte data were attenuation-corrected.

Supplementary Data Editorial assessment report.

## Data Availability

The data presented in this study are publicly available on Dryad (10.7272/Q6XK8CS6).

## Code availability

The code used to generate the findings of this study is publicly available on Zenodo (10.5281/zenodo.7098082).
