# Supplementary material for: Norepinephrine links astrocytic activity to regulation of cortical state
Source: Nat Neurosci. 2023 Mar 30;26(4):579–93. doi: 10.1038/s41593-023-01284-w (PMC10089924; doi:10.1038/s41593-023-01284-w)
Supplement: Supplementary file 1 — Supplementary Tables 1–5. [file 41593_2023_1284_MOESM1_ESM.pdf]

nature neuroscience

Article https://doi.org/10.1038/s41593-023-01284-w

Norepinephrine links astrocytic activity to regulation of cortical state

In the format provided by the
authors and unedited

Supplementary Tables:

Supplementary Table 1: Statistics for Fig. 1d

Bin X	Bin Y	Lower bound	Difference	Upper bound	p-value	Bin #	# samples
1	2	-240.15	-35.43	169.3	1.00E+00	1	249
1	3	-295.35	-90.42	114.5	9.29E-01	2	604
1	4	-399.03	-184.07	30.89	1.70E-01	3	600
1	5	-551.53	-324.93	-98.33	2.45E-04	4	447
1	6	-810.7	-569.21	-327.71	1.79E-07	5	341
1	7	-1223.7	-965.97	-708.21	1.79E-07	6	258
1	8	-1464.6	-1181.2	-897.87	1.79E-07	7	201
1	9	-1709.1	-1372	-1034.8	1.79E-07	8	146
1	10	-1955.5	-1502.1	-1048.6	1.79E-07	9	88
2	3	-211.68	-55	101.69	9.84E-01	10	42
2	4	-318.25	-148.64	20.96	1.46E-01		
2	5	-473.63	-289.5	-105.37	2.88E-05		
2	6	-735.96	-533.78	-331.6	1.79E-07		
2	7	-1151.9	-930.54	-709.19	1.79E-07		
2	8	-1396.5	-1145.8	-895.1	1.79E-07		
2	9	-1646.7	-1336.5	-1026.4	1.79E-07		
2	10	-1900.4	-1466.6	-1032.8	1.79E-07		
3	4	-263.49	-93.64	76.2	7.70E-01		
3	5	-418.86	-234.5	-50.15	2.31E-03		
3	6	-681.16	-478.78	-276.4	1.79E-07		
3	7	-1097.1	-875.55	-654.01	1.79E-07		
3	8	-1341.7	-1090.8	-839.94	1.79E-07		
3	9	-1591.8	-1281.5	-971.23	1.79E-07		
3	10	-1845.5	-1411.6	-977.75	1.79E-07		
4	5	-336.31	-140.86	54.59	4.02E-01		
4	6	-597.68	-385.14	-172.6	5.36E-07		
4	7	-1012.8	-781.9	-551.04	1.79E-07		
4	8	-1256.3	-997.15	-738.03	1.79E-07		
4	9	-1504.9	-1187.9	-870.87	1.79E-07		
4	10	-1756.7	-1318	-879.27	1.79E-07		
5	6	-468.58	-244.28	-19.97	2.03E-02		
5	7	-882.77	-641.04	-399.31	1.79E-07		
5	8	-1125.1	-856.29	-587.44	1.79E-07		
5	9	-1372.1	-1047	-722	1.79E-07		
5	10	-1621.7	-1177.1	-732.59	1.79E-07		
6	7	-652.51	-396.76	-141.02	4.03E-05		
6	8	-893.54	-612.02	-330.49	1.79E-07		
6	9	-1138.3	-802.75	-467.17	1.79E-07		
6	10	-1385.2	-932.85	-480.54	1.79E-07		
7	8	-510.85	-215.25	80.35	3.86E-01		

7	9	-753.46	-405.99	-58.52	8.32E-03
7	10	-997.29	-536.09	-74.89	8.92E-03
8	9	-557.59	-190.74	176.12	8.26E-01
8	10	-796.81	-320.84	155.14	5.04E-01
9	10	-639.92	-130.1	379.72	9.99E-01

Supplementary Table 2: Statistics for Fig. 2h

Std Threshold X	Std Threshold Y	Lower bound	Difference	Upper bound	p-value	Std Threshold	# samples
0.5	1	-2386.5	-2140.6	-1894.8	2.07E-08	0.5	5551
0.5	1.5	-3965.4	-3671.2	-3376.9	2.07E-08	1	3441
0.5	2	-4833.4	-4465.9	-4098.4	2.07E-08	1.5	2023
0.5	2.5	-5737.5	-5256.7	-4775.8	2.07E-08	2	1147
0.5	3	-7316.4	-6926.3	-6536.2	2.07E-08	2.5	617
1	1.5	-1848	-1530.5	-1213.1	2.07E-08	3	995
1	2	-2711.6	-2325.3	-1938.9	2.07E-08		
1	2.5	-3611.4	-3116	-2620.6	2.07E-08		
1	3	-5193.5	-4785.7	-4377.8	2.07E-08		
1.5	2	-1213.6	-794.74	-375.91	9.70E-07		
1.5	2.5	-2106.6	-1585.5	-1064.4	2.07E-08		
1.5	3	-3693.9	-3255.1	-2816.4	2.07E-08		
2	2.5	-1356.5	-790.76	-225.03	9.61E-04		
2	3	-2951.3	-2460.4	-1969.5	2.07E-08		
2.5	3	-2250.3	-1669.6	-1089	2.07E-08		

Supplementary Table 3: Statistics for Fig. 2j

Prolonged

Std. Threshold	# samples	p-value
0.5	5551	7.40E-03
1	3441	3.45E-01
1.5	2023	5.21E-01
2	1147	4.52E-02
2.5	617	3.14E-03
3	995	6.38E-11

Immediate

Std. Threshold	# samples	p-value
0.5	5551	9.09E-01
1	3441	4.53E-06
1.5	2023	6.30E-03
2	1147	5.70E-06
2.5	617	6.46E-08
3	995	2.25E-06

Supplementary Table 4: Statistics for Fig. 4d

Condition X	Condition Y	Lower bound	Difference	Upper bound	p-value	Condition	# samples
Sb	St	-55.885	43.189	142.26	8.16E-01	Sb	213
Sb	CNO1b	-314.17	-221.1	-128.02	2.08E-08	St	129
Sb	CNO1t	-226.4	-142.04	-57.68	2.36E-05	CNO1b	159
Sb	CNO5b	-172.33	-88.142	-3.9574	3.39E-02	CNO1t	231
Sb	CNO5t	-218.46	-115.4	-12.349	1.78E-02	CNO5b	233
St	CNO1b	-369.51	-264.28	-159.06	2.07E-08	CNO5t	114
St	CNO1t	-282.83	-185.23	-87.62	9.68E-07		
St	CNO5b	-228.79	-131.33	-33.873	1.71E-03		
St	CNO5t	-272.74	-158.59	-44.438	1.06E-03		
CNO1b	CNO1t	-12.451	79.057	170.57	1.36E-01		
CNO1b	CNO5b	41.606	132.95	224.3	4.81E-04		
CNO1b	CNO5t	-3.2907	105.69	214.68	6.34E-02		
CNO1t	CNO5b	-28.556	53.897	136.35	4.25E-01		
CNO1t	CNO5t	-75.008	26.636	128.28	9.76E-01		
CNO5b	CNO5t	-128.76	-27.261	74.239	9.73E-01		

Legend
Sb: Saline – Baseline period
St: Saline – Treatment period
CNO1b: 1mg/Kg CNO – Baseline period
CNO1t: 1mg/Kg CNO – Treatment period
CNO5b: 5mg/Kg CNO – Baseline period
CNO5t: 5mg/Kg CNO – Treatment period

Supplementary Table 5: Statistics for Fig. 4f

Condition X	Condition Y	Lower bound	Difference	Upper bound	p-value	Condition	# samples
Sb	St	-18.115	99.152	216.42	1.53E-01	Sb	126
Sb	CNO1b	-316.41	-191.86	-67.3	1.65E-04	St	264
Sb	CNO1t	-216.34	-98.561	19.221	1.62E-01	CNO1b	189
Sb	CNO5b	-180.52	-63.747	53.029	6.28E-01	CNO1t	257
Sb	CNO5t	-403.61	-281.46	-159.31	2.14E-08	CNO5b	271
St	CNO1b	-394.2	-291.01	-187.82	2.07E-08	CNO5t	209
St	CNO1t	-292.62	-197.71	-102.81	6.33E-08		
St	CNO5b	-256.55	-162.9	-69.247	1.06E-05		
St	CNO5t	-480.88	-380.61	-280.34	2.07E-08		
CNO1b	CNO1t	-10.481	93.296	197.07	1.07E-01		
CNO1b	CNO5b	25.475	128.11	230.74	5.04E-03		
CNO1b	CNO5t	-198.31	-89.601	19.108	1.75E-01		
CNO1t	CNO5b	-59.483	34.814	129.11	9.00E-01		
CNO1t	CNO5t	-283.77	-182.9	-82.022	3.56E-06		
CNO5b	CNO5t	-317.41	-217.71	-118.01	2.77E-08		

Legend
Sb: Saline – Baseline period
St: Saline – Treatment period
CNO1b: 1mg/Kg CNO – Baseline period
CNO1t: 1mg/Kg CNO – Treatment period
CNO5b: 5mg/Kg CNO – Baseline period
