# Supplementary material for: Norepinephrine links astrocytic activity to regulation of cortical state
Source: Nat Neurosci. 2023 Mar 30;26(4):579–93. doi: 10.1038/s41593-023-01284-w (PMC10089924; doi:10.1038/s41593-023-01284-w)
Supplement: Supplementary file 2 — Reporting Summary [file 41593_2023_1284_MOESM2_ESM.pdf]

nature research

Corresponding author(s): Kira Poskanzer

Last updated by author(s): Dec 24, 2022

Reporting Summary

Nature Research wishes to improve the reproducibility of the work that we publish. This form provides structure for consistency and transparency in reporting. For further information on Nature Research policies, see our Editorial Policies and the Editorial Policy Checklist.

Statistics

For all statistical analyses, confirm that the following items are present in the figure legend, table legend, main text, or Methods section.

n/a	Confirmed	
☐	☒	The exact sample size (*n*) for each experimental group/condition, given as a discrete number and unit of measurement
☐	☒	A statement on whether measurements were taken from distinct samples or whether the same sample was measured repeatedly
☐	☒	The statistical test(s) used AND whether they are one- or two-sided<br>*Only common tests should be described solely by name; describe more complex techniques in the Methods section.*
☐	☒	A description of all covariates tested
☐	☒	A description of any assumptions or corrections, such as tests of normality and adjustment for multiple comparisons
☐	☒	A full description of the statistical parameters including central tendency (e.g. means) or other basic estimates (e.g. regression coefficient) AND variation (e.g. standard deviation) or associated estimates of uncertainty (e.g. confidence intervals)
☐	☒	For null hypothesis testing, the test statistic (e.g. *F*, *t*, *r*) with confidence intervals, effect sizes, degrees of freedom and *P* value noted<br>*Give P values as exact values whenever suitable.*
☒	☐	For Bayesian analysis, information on the choice of priors and Markov chain Monte Carlo settings
☒	☐	For hierarchical and complex designs, identification of the appropriate level for tests and full reporting of outcomes
☐	☒	Estimates of effect sizes (e.g. Cohen's *d*, Pearson's *r*), indicating how they were calculated

Our web collection on statistics for biologists contains articles on many of the points above.

Software and code

Policy information about availability of computer code

Data collection	Imaging data and associated electrical data was collected using PrairieView software (version 5). Electrical recordings without imaging were acquired using PackIO. Pupil recordings were acquired through MATLAB Image Acquisition toolbox.
Data analysis	Data analysis was done using AQuA (1), Suite2P (2018), Chronux (2.12), and standard analysis was done in MATLAB (2020a) and Python (v3). The code that was used to generate the findings of this study are publicly available on Zenodo (DOI: 10.5281/zenodo.7098082).

For manuscripts utilizing custom algorithms or software that are central to the research but not yet described in published literature, software must be made available to editors and reviewers. We strongly encourage code deposition in a community repository (e.g. GitHub). See the Nature Research guidelines for submitting code & software for further information.

Data

Policy information about availability of data

All manuscripts must include a data availability statement. This statement should provide the following information, where applicable:

- Accession codes, unique identifiers, or web links for publicly available datasets
- A list of figures that have associated raw data
- A description of any restrictions on data availability

The datasets generated during the current study are available on Dryad (DOI: 10.7272/Q6XK8CS6).

Field-specific reporting

Please select the one below that is the best fit for your research. If you are not sure, read the appropriate sections before making your selection.

☒ Life sciences ☐ Behavioural & social sciences ☐ Ecological, evolutionary & environmental sciences

For a reference copy of the document with all sections, see nature.com/documents/nr-reporting-summary-flat.pdf

Life sciences study design

All studies must disclose on these points even when the disclosure is negative.

Sample size	No statistical methods were used to predetermine sample size, but our sample sizes are similar to those reported in previous publications (Bojarskaite, 2020; Ding, 2019; Paukert, 2014; Reimer, 2014, 2016), and statistical significance was calculated using post-hoc tests.
Data exclusions	No data was excluded from analyses except for the following (not pre-determined): In hSYN-hM4Di experiments, outliers were excluded across all conditions from small stationary responses to avoid confounding effects from other influences on astrocyte Ca2+, as described in methods. For in vivo pharmacology experiments, electrical artifacts in band power were excluded before analysis, as described in methods.
Replication	Empirical findings were replicated across multiple animals and multiple days and were successful. Replications are listed in each figure. Random Forest Regression accuracy was confirmed using ten cross-validations. In analysis of hSYN-hM4Di effects on event rate, resampling was performed ten thousand times to confirm accuracy and the distribution of results is plotted in the figure. Two-photon imaging at different acquisition rates was performed in the same mice in one separate experiment and confirmed our findings in astrocyte KO conditions. Multiple image analysis methods were used to confirm GRAB-NE results (Two-photon and fiber photometry) with separate cohorts of mice.
Randomization	Samples were randomly allocated into experimental groups by cell-type expression of each individual fluorescent sensor, and ex vivo or in vivo methodology. Only adult animals (1-6 months of age) were used in experiments, and both male and female were used and randomly selected.
Blinding	For imaging and electrical recordings of spontaneous activity, blinding was not relevant because cell-type viral expression is evident from expression pattern. For in vivo pharmacology, blinding was not possible because control recordings were taken prior to treatment recordings to avoid confounding the treatment effects. For Adra1A floxed mice, the experimenter was blinded to genotype before data collection and analysis.

Reporting for specific materials, systems and methods

We require information from authors about some types of materials, experimental systems and methods used in many studies. Here, indicate whether each material, system or method listed is relevant to your study. If you are not sure if a list item applies to your research, read the appropriate section before selecting a response.

Materials & experimental systems

n/a	Involved in the study	
☐	☒	Antibodies
☐	☒	Eukaryotic cell lines
☒	☐	Palaeontology and archaeology
☐	☒	Animals and other organisms
☒	☐	Human research participants
☒	☐	Clinical data
☒	☐	Dual use research of concern

Methods

n/a	Involved in the study	
☒	☐	ChIP-seq
☒	☐	Flow cytometry
☒	☐	MRI-based neuroimaging

Antibodies

Antibodies used	Chicken α-GFP (1:3000, Abcam, ab13970), rabbit α-NeuN (1:1000, EMD Millipore, ABN78), mouse α-NeuN (1:1000, Millipore Sigma, MAB377) and rabbit α-S100B (1:500, Millipore Sigma, SAB5500172) were used in this study.
Validation	All antibodies used in this study were validated using at least western blot and/or immunocytochemistry by the manufacturer. All antibodies used in this study have been used in previous studies and referenced by the manufacturer.

Eukaryotic cell lines

Policy information about cell lines

Cell line source(s)	JM8 and JM8.F6 sublines were derived from C57BL/6N mice.

Authentication	None of the cell lines used were authenticated.
Mycoplasma contamination	All cell lines tested negative.
Commonly misidentified lines (See ICLAC register)	None.

Animals and other organisms

Policy information about studies involving animals; ARRIVE guidelines recommended for reporting animal research

Laboratory animals	Adult mC57BL/6 mice, Adra1a fl/fl mice, and Adra1a wild-type mice (mixed males and females) were used in this study as indicated. All mice were adults (aged 1–6 months) at time of surgery. Animal housing rooms were kept at 68-74 degrees Fahrenheit and 30-70% humidity.
Wild animals	The study did not involve wild animals
Field-collected samples	The study did not involve samples collected from the field
Ethics oversight	All experimental procedures were approved by the UCSF Institutional Animal Care and Use Committee.

Note that full information on the approval of the study protocol must also be provided in the manuscript.
