# Supplementary material for: Norepinephrine links astrocytic activity to regulation of cortical state
Source: Nat Neurosci. 2023 Mar 30;26(4):579–93. doi: 10.1038/s41593-023-01284-w (PMC10089924; doi:10.1038/s41593-023-01284-w)
Supplement: Supplementary file 7 — Editorial assessment report. [file 41593_2023_1284_MOESM7_ESM.pdf]

nature portfolio GUIDED OPEN ACCESS

Contents of this report

- **Manuscript details**: overview of your manuscript and the editorial team.
- **Review synthesis**: summary of the reviewer reports provided by the editors.
- **Editorial recommendation**: personalized evaluation and recommendation from all 3 journals.
- **Annotated reviewer comments**: the referee reports with comments from the editors.
- **Open research evaluation**: advice for adhering to best reproducibility practices.

About the editorial process

Because you selected the **Nature Portfolio Guided Open Access option**, your manuscript was assessed for suitability in three of our titles publishing high-quality work across your field of research. More information about Guided Open Access can be found **here**.

Collaborative editorial assessment

Your editorial team discussed the manuscript to determine its suitability for the Nature Portfolio Guided OA pilot. Our assessment of your manuscript takes into account several factors, including whether the work meets the **technical standard** of the Nature Portfolio and whether the findings are of **immediate significance** to the readership of at least one of the participating journals in the Guided OA pilot.

Peer review

Experts were asked to evaluate the following aspects of your manuscript:

- **Novelty** in comparison to prior publications;
- **Likely audience** of researchers in terms of broad fields of study and size;
- **Potential impact** of the study on the immediate or wider research field;
- **Evidence** for the claims and whether additional experiments or analyses could feasibly strengthen the evidence;
- **Methodological detail** and whether the manuscript is reproducible as written;
- Appropriateness of the literature review.

Editorial evaluation of reviews

Your editorial team discussed the potential suitability of your manuscript for each of the participating journals. They then discussed the revisions necessary in order for the work to be published, keeping each journal's specific editorial criteria in mind.

Journals in the Nature portfolio will support authors wishing to transfer their reviews and (where reviewers agree) the reviewers' identities to journals outside of Springer Nature.

If you have any questions about review portability, please contact our editorial office at guidedoa@nature.com.

nature portfolio GUIDED OPEN ACCESS

Manuscript details

Tracking number	Submission date	Decision date
GUIDEDOA-21-00114	4 May 2021	30 June 2021

**Title**	Norepinephrine links astrocytic activity to regulation of cortical state	**Corresponding author**	Kira Poskanzer<br>**Affiliation:** University of California, San Francisco
**Preprint information**	No preprint is available for this manuscript.	**Peer review type**	Single-blind

Editorial assessment team

**Primary editor**	**Luis Mejia**<br>**Home Journal:** *Nature Neuroscience*, ORCID: 0000-0001-5439-6803<br>**Email:** luis.mejia@us.nature.com
**Editorial team members**	**David Rowland**, *Nature*, ORCID: 0000-0002-2735-2730<br>**Christian Schnell**, *Nature Communications*, ORCID: 0000-0002-3499-9217
**About your primary editor**	Luis joined Nature Neuroscience in 2018. He received his Ph.D. in Neuroscience from Harvard University/Harvard Medical School, followed by postdoctoral research at Cold Spring Harbor Laboratory in the lab of Bo Li, where he investigated orbitofrontal-striatal projection neurons in value and valence based decisions and behaviors in mice, using in vivo optogenetics and calcium imaging. His research interests include systems and circuits neuroscience, reward and aversion learning, and in vivo imaging and neuroscience methods. Luis is based in the New York office.

Editorial assessment and review synthesis

**Editor's summary and assessment**	The authors examine the role of astrocytic activity in norepinephrine- and arousal-regulated changes in cortical state (desynchrony, restoration of synchrony). They perform astrocyte-specific (or neuron-specific) calcium imaging and NE indicator imaging in visual cortex in awake mice. The findings suggest that arousal and NE desynchronize cortical activity, but this is followed by restoration of synchrony via activation of astrocytic alpha 1-NE receptors (negative feedback mechanism). The imaging results place the astrocyte calcium activity upstream of the neuronal activity.<br><br>The editors found the work linking NE and arousal/state transitions to astrocytic activity and cortical synchrony to be of interest, and technically well executed with dual-color imaging and simultaneous imaging and LFP recordings. The editors also felt that additional functional insight with manipulations and behavior would be desirable, and noted that some of the separate findings were confirmatory, though novelty was deemed sufficient for external review.<br><br>As part of the Guided Open Access pilot, editors from *Nature*, *Nature Neuroscience* and *Nature Communications* have discussed the reviewer reports and the manuscript's suitability for the journals. After careful evaluation, our editorial recommendation is to revise the manuscript and submit back through the Guided Open Access submission portal for consideration at *Nature Neuroscience* or *Nature Communications*.
**Editorial synthesis of reviews**	Your manuscript has been seen by 3 reviewers with expertise in state-dependent cortical activity, in vivo imaging, astrocytes and neuron-glia interactions, and neuromodulation. While the reviewers find the work of interest, they have raised substantial concerns about technical aspects of the study as well as the data support for the conclusions and the advance.<br><br>To be considered further at *Nature Neuroscience* you would need to address all the reviewer concerns, including the technical points regarding statistical analyses and number of animals, hemodynamic correction, alternative interpretations and controls for the imaging results, as well as the request to image astrocyte and neuron activity in Adra1a cKO mice to strengthen the support for the conclusions regarding astrocyte responses and synchrony.<br><br>*Nature Communications* would be prepared to consider a revised version that addresses the reviewers' technical and statistical concerns with additional data/analyses. They do not consider the additional experiments required to address Ref #2's point 1 (imaging of neurons and astrocytes in Adra1a floxed mice) essential.

Editorial recommendation

Journal	Recommendation	Comments
**Nature**	**Revision not invited**	Following editorial assessment of the paper and reviewer reports it was felt that the conceptual advance is not sufficient for further consideration at *Nature*.
**Nature Neuroscience**	**Major revisions with extension of the study**	The editors would expect to see all major points addressed with additional data/analyses. This would include increasing the number of animals and resolving the statistical analysis concern as raised by Ref #1 point 1, and providing controls for hemodynamic correction (in particular for the NE indicator imaging) as raised by Ref #1 point 2. In addition, we feel that further characterization of the astrocytic and neuron calcium responses in Adra1a cKO mice would be important to strengthen the support for the conclusions, as raised by Ref #2 point 1, as is resolving the questions regarding alternative interpretations and controls for the imaging results, as raised by Ref #3 points 1-5, and the scope of conceptual advance in the findings as alluded to by Ref #3 point 3.
**Nature Communications**	**Major revisions**	The editors find that the authors need to address most of the reviewers' comments with additional data/analyses, including increasing the number of animals and resolving the statistical concern as raised by Ref #1 point 1, providing imaging controls for hemodynamic correction (in particular for the NE indicator imaging) as raised by Ref #1 point 2, providing controls for the imaging results, as raised by Ref #3 points 1-5, and discussing the scope of conceptual advance in the present findings as alluded to by Ref #3 point 3. They do not consider the additional experiments required to address Ref #2 major point 1 essential.

Next steps

Recommendation Summary

- Option 1: Revise for consideration at Nature Neuroscience
- Option 2: Revise for consideration at Nature Communications

See the previous page for details

Revision

If you would like to follow our recommendation, please upload the revised manuscript, along with your point-by-point response to the reviewers' reports and editorial advice **using the link provided in the decision letter**.

Revision checklist

- Cover letter, stating to which journal you are submitting
- Revised manuscript
- Point-by-point response to reviews
- Updated **Reporting Summary** and **Editorial Policy Checklist**
- Supplementary materials (if applicable)

Submission elsewhere

To a journal outside of Nature Portfolio

We can share the reviews with another journal outside of the Nature Portfolio if requested. You will need to request that the receiving journal office contacts us at guidedOA@nature.com. We have included editorial guidance below in the reviewer reports and open research evaluation to aid in revising the manuscript for publication elsewhere.

Annotated reviewer reports

The editors have included some additional comments on specific points raised by the reviewers below, to clarify requirements for publication in the recommended journal(s). However, please note that all points should be addressed in a revision, even if an editor has not specifically commented on them.

Reviewer #1	
**Reviewer #1**	This reviewer has not chosen to waive anonymity. The reviewer's identity can only be shared with representatives of an established journal editorial office.
**Reviewer #1 expertise**<br>Summarized by the editor	State-dependent cortical activity, in vivo imaging and behavior, neuromodulation
**Editor's comments about this review**	The reviewer has provided an overall positive assessment of the paper, but has raised important concerns regarding the technical aspects to support the conclusions.
**Reviewer #1 comments**	
**Overview**	This is an impressive study using a series of different tools and approaches to examine the role of NE in the regulation of astrocyte activity and cortical state. Intriguingly, the authors find that astrocyte activity is robustly modulated by state transitions, rather than steady-state periods of arousal or quiescence. They present some evidence that the state-dependent changes in astrocyte calcium signaling are mediated by NE via the a1A-NE receptor specifically on astrocytes.<br>Overall, this is an interesting topic and builds on a large body of work highlighting the dynamics and mechanisms underlying state-dependent cortical regulation. The findings will be of interest to broad subsets of the field.

Specific comments

#	Reviewer comment	Editorial comment
1	Statistics are a major concern for this study in its current form. Throughout the manuscript, it appears that cells or sessions have been used as the n for statistical comparison. This is entirely inappropriate, given the nested experimental design. The authors should either use animals as the n for each test or use an appropriate hierarchical analytical approach (see Saravanan, Berman, and Sober 2020 as an example). The number of animals is quite low for some of the datasets (4 or 5 mice in some cases), so more experiments may be necessary to perform appropriate statistical tests.	Nature Neuroscience and Nature Communications would ask that you resolve the statistical concerns and increase the number of animals.
2	A second concern centers on the authors' approach for estimating hemodynamic contamination in the imaging data for the GRABSne. The authors are making assumptions about the absorption coefficient and the path length that are not able to be validated for this approach. In addition, it is unclear what the ROI size is for the signal they are analyzing here. If the ROIs are small, then hemodynamic correction is not necessary. If the ROIs are large, then the authors should add new experimental data to validate the correction using appropriate controls. Along these lines, if the ROIs are large, it is unclear why the authors chose to use 2-photon imaging for these data. 1-photon imaging would provide more robust signals for this tool, which has relatively poor 2-photon signal quality, and would allow the authors to use a more accepted and well validated approach for hemodynamic correction, such as backscatter illumination.	Nature Neuroscience and Nature Communications would ask that you address the technical concerns regarding hemodynamic correction in the imaging.

Reviewer #2

**Reviewer #2**	This reviewer has not chosen to waive anonymity. The reviewer's identity can only be shared with representatives of an established journal editorial office.

**Reviewer #2 expertise**<br>Summarized by the editor	Astrocytes and neuronal activity, in vivo imaging and behavior
**Editor's comments about this review**	The reviewer has provided an overall positive assessment of the paper, but raises important comments regarding the strength of the conclusions about the astrocytic responses and relation to NE and synchrony.

Reviewer #2 comments

**Overview**	Reitman et al, in their manuscript, show that astrocyte activity is correlated with changes in arousal (correlated with pupil relative change, not pupil diameter of speed), and this correlation is apparent also in stationary state. NE, on the other hand, better represents arousal level (not changes in arousal). Astrocytic response to NE is larger and longer the highest the phasic NE peak is. Astrocytes Ca happens before neuronal Ca following arousal. Then, as if dual imaging of Ca events is not complicated enough, the performed dual (ipsi- and contra- lateral) electrophysiology… They found that astrocytic activity is correlated not only with local neuronal activity, bot with ipsi-, and even contra-lateral LFP activity: low frequency goes up, and high frequency goes down before astrocyte Ca changes, but these differences are gone with the addition of prazosin (alpha1NE-receptor inhibitor). The A61603, an alpha1NE agonist, caused an increase in LFP (LF only), increased both neuronal and astrocytic Ca, and elevated their synchronization. Finally, they generated a mouse line allowing deletion of alpha1NE from astrocytes. These mice have higher HF and lower LF during movement. In summary, they show that except from the desynchronizing (know) effect of NE, it also has a synchronizing effect via astrocytes.<br><br>The paper is a technical tour de force, employing many tools (dual population imaging, AQuA, LFP recording, smFISH, DREADDs…) sometimes together… and is well written. Their interpretation of the result makes sense to me.

Specific comments

#	Reviewer comment	Editorial comment
**1**	1) The main point of the paper is that astrocytes mediate a synchronizing effect of NE. But from all the points, it's the least proved… I think a warranted experiment would be to image neurons and astrocytes in Adra1A floxed mice, and assuming NE affects astrocytes, which in turn affect	Nature Neuroscience would encourage you to add this imaging experiment in Adra1A cKO mice, in support of the strength of the conclusions. Nature

	neurons, I would expect: 1) Astro will show less (if at all) response to NE. 2) Astrocytes will no longer be active before the neurons.	Communications would not consider this experiment to be essential.
**2**	2) This paper has A LOT of data in it. I would consider to have less figures than now (for example 3+4 and 5+6 can easily be joined), and some data can go to the supplement, and make the paper easier to follow. However, I leave it to the authors to decide how to make the paper clearer (graphically).	
**3**	Minor: I couldn't find a reference to Extended Data fig. 5 in the main text.	

**Reviewer #3**	
**Reviewer #3**	This reviewer has not chosen to waive anonymity. The reviewer's identity can only be shared with representatives of an established journal editorial office.
**Reviewer #3 expertise**<br>Summarized by the editor	Astrocytes and neuron-glia interactions, in vivo imaging and behavior, neuromodulation
**Editor's comments about this review**	The reviewer has provided an overall positive assessment of the paper, but raises important questions/clarifications and concerns regarding the interpretations and strength of the conclusions in the imaging results, as well as on the scope of insight in the present findings.
**Reviewer #3 comments**	
**Overview**	The manuscript "Norepinephrine links astrocytic activity to regulation of cortical state" by Reitman et al. is well written. It introduces a model that places astrocytes in a central role for awake cortical state regulation, acting as a negative feedback mechanism for arousal-associated desynchrony.<br>The central hypothesis of this manuscript is to test whether astrocytic NE signaling acts as a distinct neuromodulatory pathway, regulating cortical states, and linking arousal-associated desynchrony to cortical circuit resynchronization. Using state-of-the-art in vivo two-Photon imaging, imaging analyses, electrophysiological, viral transduction, and genetic approaches, the authors demonstrate that NE-signaling to astrocytes plays a crucial role in astrocytes regulating cortical states arousal-

	associated desynchrony and cortical resynchronization. Overall, this study lays a foundation that shows how astrocytes can be an extension of the NE modulatory network. Thus, this study does have the potential to move the glia field forward. However, I do have several critiques and comments.

Specific comments

#	Reviewer comment	Editorial comment
**1**	1. Are the authors concern with potential adaptions of signals in astrocytes and neurons due to pupil diameter changes that could influence their interruption of data?	Nature Neuroscience and Nature Communications would expect that you appropriately resolve all the technical concerns regard interpretation and alternative explanations.
**2**	2. In Figure 2D, the authors stated that the maximum cross-correlation was broad and continued for at least 20s after the pupil diameter, reflecting the persistence of extracellular NE. However, NE was already high before pupil diameter. Why are the authors convinced that pupil diameter caused a persistent increase in NE? It would be nice to see a control that shows levels fall in the absence of pupil diameter. I think the authors need to provide some clarity for this rationale.	Nature Neuroscience and Nature Communications would expect that you appropriately resolve the technical concerns regard interpretation and alternative explanations.
**3**	3. In Figure 3, the authors conclude that their results support a model in which arousal drives astrocytic Ca2+, which leads to increases in Ca2+ activity and synchrony of nearby neurons. Could the authors add more details or speculate on just how rises in NE-mediated astrocytic Ca2+ is causing Ca2+ increases and synchrony of nearby neurons?	Nature Neuroscience would expect a data- and analyses-driven response to this critique regarding the mechanistic scope of the advance.<br><br>Nature Communications would expect that you appropriately address the critique with analytical or textual discussion.
**4**	4. In the representative trace of Figure 3C, some of the neuronal Ca2+ increases are higher than the astrocytes when comparing responses of movement to Ca2+ responses.	
**5**	5. The authors used A61603 (1g/kg, i.p.) to block 1A-NE receptor. Because the authors injected this IP, how do	Nature Neuroscience and Nature Communications would expect that

	they rule out any downstream effects of A61603 that may confound the interpretation of some of their results vs. locally puffing the drug to a specific ROI? Some language should be added to the discussion or section to offer some clarification.	you appropriately resolve the technical concerns regard interpretation and alternative explanations in a data-driven manner.
**6**	Minor Comment Figure 1M is hard to read the authors may want to change their color scheme.	

Open research evaluation

Data availability

Data Availability statement

Please add a Data Availability statement. Please ensure that your Data Availability statement includes accession details for deposited data, mentions where Source data can be found, and states that all other data are available from the corresponding author (or other sources, as applicable) on reasonable request. More information about our data availability policy can be found here: https://www.nature.com/nature-portfolio/editorial-policies/reporting-standards#availability-of-data
See here for more information about formatting your Data Availability Statement: http://www.springernature.com/gp/authors/research-data-policy/data-availability-statements/12330880

Thank you for including a Data Availability statement. However, we noted that you have only indicated that data are available upon request. The data availability statement must make the conditions of access to the “minimum dataset” that are necessary to interpret, verify and extend the research in the article, transparent to readers.
In addition, Nature Portfolio policies include a strong preference for research data to be archived in public repositories. For data types without specific repositories, we recommend that data are deposited in a generalist repository such as figshare or Dryad. More information about our data availability policy can be found here: https://www.nature.com/nature-portfolio/editorial-policies/reporting-standards#availability-of-data
See here for more information about formatting your Data Availability Statement: http://www.springernature.com/gp/authors/research-data-policy/data-availability-statements/12330880

Other data requests

All source data underlying the graphs and charts presented in the main figures should be made available as Supplementary Data (in Excel or text format) or via a generalist repository (eg, Figshare or Dryad). This is strongly encouraged for publication in a Nature Portfolio journal, but is also best practice for publication in any venue.

Please ensure that a Source Data file is included with your resubmission. The Source Data file contains the raw data underlying the following types of display items:

- Any reported means/averages in box plots, bar charts, and tables

- Dot plots/scatter plots, especially when there are overlapping points

- Line graphs

- Uncropped blots and gel images, pasted in and labelled with the relevant panel and identifying information such as the antibody used.

The data should be provided in a single Excel file with data for each figure/table in a separate sheet, or in multiple labelled files within a zipped folder.

The file should be labelled 'Source Data' and should be mentioned in all relevant figure legends using the template text below:
"Source data are provided as a Source Data file.". The “Data Availability” section should also include the statement “Source data are provided with this paper.”

To learn more about our motivation behind this policy, please see: https://www.nature.com/articles/s41467-018-06012-8. An example of the Source Data file is available demonstrating the correct format: https://www.nature.com/documents/ncomms-example-source-data.xlsx

Code availability and citation

Please include a statement under the heading "Code Availability", indicating whether and how the custom code/software reported in your study can be accessed, including any restrictions to access. This section should also include information on the versions of any software used, if relevant, and any specific variables or parameters used to generate, test, or process the current dataset. Code availability statements should be provided as a separate section after the Data Availability section. Upon publication, Nature Portfolio journals consider it best practice to release custom computer code in a way that allows readers to repeat the published results. Code should be deposited in a DOI-minting repository such as Zenodo, Gigantum or Code Ocean and cited in the reference list following the guidelines described in our policy pages (see link below). Authors are encouraged to manage subsequent code versions and to use a license approved by the open source initiative.
Full details about how the code can be accessed and any restrictions must be described in the Code Availability statement.
See here for more information about our code availability policies: https://www.nature.com/nature-portfolio/editorial-policies/reporting-standards#availability-of-computer-code
We also provide a Code and Software submission checklist that you may find useful: https://www.nature.com/documents/nr-software-policy.pdf
Please note: because of advanced features used in this form, you must use Adobe Reader to open the documents and fill it out.

Thank you for including a Code Availability statement. However, we noted that you have only indicated that custom code are available upon request. The code availability statement must indicate whether and how the code or algorithm can be accessed, including any restrictions to access. Upon publication,

Nature Portfolio journals consider it best practice to release custom computer code in a way that allows readers to repeat the published results. Code should be deposited in a DOI-minting repository such as Zenodo, Gigantum or Code Ocean and cited in the reference list following the guidelines described in our policy pages (see link below). Authors are encouraged to manage subsequent code versions and to use a license approved by the open source initiative. Full details about how the code can be accessed and any restrictions must be described in the Code Availability statement.
See here for more information about our code availability policies: https://www.nature.com/nature-portfolio/editorial-policies/reporting-standards#availability-of-computer-code
We also provide a Code and Software submission checklist that you may find useful: https://www.nature.com/documents/nr-software-policy.pdf
Please note: because of advanced features used in this form, you must use Adobe Reader to open the documents and fill it out.

Materials availability

We encourage you to include within the Data Availability statement whether your Adra1A floxed mice can be made available to readers. If so, please also include the email address for requests.

Ethics

Please provide a 'Competing interests' statement using one of the following standard sentences:
1. The authors declare the following competing interests: [specify competing interests]
2. The authors declare no competing interests.
See our competing interests policy for further information: https://www.nature.com/nature-research/editorial-policies/competing-interests

Because your study uses live vertebrates, a statement affirming that you have complied with all relevant ethical regulations for animal testing and research is necessary. A statement explicitly confirming if the study received ethical approval, including the name of the board and institution that approved the study protocol is also required. The species, strain, sex and age of animals should be included.

Reporting and reproducibility

Reporting

For all statistics (including error bars), please provide the EXACT n values used to calculate the statistics (reporting individual values rather than a range if n varied among experiments) AND define type of replicates (e.g., cell cultures, technical replicates). Please avoid use of the ambiguous term “biological replicates”; instead state what constituted the replicates (e.g., cell cultures, independent experiments, etc.). For all representative results, indicate number of times experiments were repeated, number of images collected, etc. Indicate statistical tests used, whether the test was one- or two-tailed, exact values for both significant and non-significant *P* values where relevant, *F* values and degrees of freedom for all ANOVAs and t-values and degrees of freedom for *t*-tests. **If this is too much information to include in the figure legends, we recommend providing it as a supplementary table and referencing the table in the statistics section.**

Reproducibility

A Word document indicating revisions that need to be made in compliance with our reporting summary is attached. The detailed comments document lists all of the changes that need to be made to the text, and particularly the main and supplementary figure legends, including (but not limited to) details regarding sample sizes, replication, scale and error bars, and statistics.

Please include a statement indicating how the sample sizes were chosen in the Methods section. If a power analysis was used, provide the details of this analysis. If you did not use a power analysis, the following is sufficient: "No statistical methods were used to pre-determine sample sizes but our sample sizes are similar to those reported in previous publications (ref x,y,z)." Stating that sample sizes were chosen to demonstrate statistically significant effects is only accurate if you performed an a priori test to determine this value.

Please indicate whether the data met the assumptions of the statistical tests used, including whether normality and equal variances were formally tested. If not, please show data distribution (individual data points) and include the following statement: "Data distribution was assumed to be normal but this was not formally tested.”

Please include a statement on randomization in the Methods. Indicate whether the data collection was randomized or appropriately blocked, how animal/samples were assigned to the various experimental groups and whether there was any randomization in the organization of the experimental conditions or stimulus presentations.

Please include a statement indicating whether blinding was used in the Methods. If there was no blinding, this must be clearly stated in the manuscript, as follows: "Data collection and analysis were not performed blind to the conditions of the experiments.”

Please disclose whether any animals or data points were excluded from the analyses for any reason and note the rationale for the exclusions.

Statistics

Error bars should be displayed wherever possible and must be clearly defined in the caption for each figure. To improve reproducibility of your analyses, please provide details regarding your treatment of outliers.

Methods descriptions

The Methods must contain sufficient detail such that the work could be replicated. It is preferable that all key methods be included in the main manuscript, rather than in the Supplementary Information.

The methods section can be around 3,000 words in length (no strict limit), they can contain references that do not count towards the reference limit in the main paper, and will be fully indexed. You should feel free, and we in fact encourage you, to incorporate any part of your Supplementary Information that you feel is important for the rest of the paper within this section.

The Methods section should be written as concisely as possible but should contain all elements necessary to allow interpretation and reproduction of the results (please note, however, that the methods section cannot contain any figures or tables at present).

If there are additional references in the Methods section, their numbering should continue from the last reference in the main paper, and the list should follow the Methods section.

Other notes

We have included as an attachment to the decision letter a version of your Reporting Summary with a few notes. This is mainly for your information, but we hope it is helpful when preparing your revised manuscript. If you decide to resubmit the manuscript for further consideration, please be sure to include an updated Reporting Summary.
